# Ammonification Process Is Limited by Transformation of Medium Molecular Weight Nitrogenous Organics but Not Hydrolysis of Proteins

**DOI:** 10.1002/advs.77787

**Published:** 2026-09-16

**Authors:** Jing Li, Junjie Qiu, Fan Lü, Hua Zhang, Pinjing He

**Affiliations:** ^1^ Institute of Waste Treatment and Reclamation, College of Environmental Science and Engineering Tongji University Shanghai People's Republic of China; ^2^ State Key Laboratory of Water Pollution Control and Green Resource Recycling, College of Environmental Science and Engineering Tongji University Shanghai People's Republic of China; ^3^ Shanghai Institute of Pollution Control and Ecological Security Shanghai People's Republic of China

**Keywords:** nitrogen‐cycle, non‐targeted analysis, organic nitrogen transformation, proteomics

## Abstract

Ammonification is a critical step in nitrogen cycling framework, but the role of molecular heterogeneity within dissolved organic nitrogen (DON) remains elusive. A controlled laboratory experiment across redox regimes resolved stepwise conversion of DON from proteins to small nitrogenous intermediates. Integrated datasets from macromolecules to monomers from a steady reactor overcame challenging in impossible quantification of all DON compounds in the kinematic research. Data‐independent based proteomic analysis revealed that over 85% of proteins remained stable under anaerobic and microaerobic conditions. Non‐targeted profiling and paired‐mass‐difference analysis revealed that partial transformations of DON did not cover nitrogen‐containing functional groups. Peptide‐level results identified methionine‐ and tryptophan‐rich sequences as the persistent constituents, as well as theoretical analysis of frontier molecular orbital. Targeted quantification of DON monomers presented low concentrations of amino acids and purine derivatives, reflecting rapid and nearly complete transformation thereof. Thereby, ammonification was constrained by the formation and subsequent transformation of medium‐molecular‐weight nitrogenous intermediates instead of protein hydrolysis process. These findings underscore the structural constraints governing nitrogen mineralization as a new mechanism of DON transformation.

## Graphical abstract

A multi‐level analytical framework, integrating protein‐level persistence analysis, nontargeted HRMS formula tracking, peptide‐level proteomics, and targeted monomer quantification under varying redox conditions, together with FMO calculations reveal that ammonification is limited by medium molecular weight nitrogenous multimers. It provides a transferable approach for characterizing DON transformation across systems of varying substrate origins.

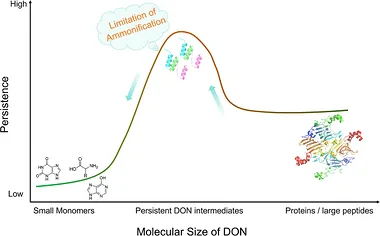

## 1 Introduction

Ammonification is catalytical deamination reactions of dissolved organic nitrogen (DON) in liquid phase, referring to the degradation of both readily biodegradable and recalcitrant DON fractions to yield ammonia, which microorganisms utilize for growth and metabolism [1]. Deamination of DON represents a major source of dissolved inorganic nitrogen regeneration in the ocean [2, 3] and a fundamental mechanism of nutrient recycling in synthetic ecosystems [4]. At the sediment‐water interface of estuaries, deamination of DON would contribute a large amount of inorganic nitrogen to bottom water [5], and oxidative transformation of DON in marginal seas can produce carboxyl‐rich alicyclic molecules as by‐products [6]. In wastewater treatment plants, exogenous DON, including proteins and amino acids (AAs) [7], is removed through microbial metabolism during anaerobic treatment [8, 9], yet the chemical composition of effluent DON varies with influent characteristics and microbial activity [10]. Collectively, the interconversion between ammonia and organic nitrogen constitutes one of the largest nitrogen fluxes in both natural and engineered systems [11].

The classic model of ammonification posits that proteins are first hydrolyzed by extracellular enzymes, followed by AAs liberating ammonia primarily via Stickland reactions [12]. The hydrolysis step is generally considered as the limitation in the stepwise conversion. However, owing to molecular diversity within the DON pool, the structure of DON significantly affects its transformation pathways and barrier of ammonification. Aromatic AAs (e.g., tryptophan, tyrosine), readily release ammonium under light exposure, whereas nucleotides and urea exhibit minimal conversion [13]. Thus, bulk analysis of DON failed to resolve the abovementioned obscurities towards ammonification of DON [14, 15]. Even though anaerobic process could conduct the hydrolysis of DON [16] and micro‐aeration can indeed accelerate the ammonification [17, 18, 19], higher concentration of ammonium [20] by enhanced ammonification would depress the degradation of DON with stable chemical properties. Consequently, the structure‐dependent reactivity of DON conversion under varying redox conditions remains elusive.

Common compounds of DON involve proteins, peptides, AAs, purines and pyrimidines. Whereas, it remains impossible to conduct stepwise kinetic analyses according to the realistic mass concentrations of these compounds due to current methodological limitation. Several analytical approaches have been developed to characterize DON transformation across different molecular scales. Small molecules like AAs, purines and pyrimidines with a stable chemical structure could be quantitated by target analysis using liquid chromatography with tandem mass spectrometry. Non‐targeted analysis by using high‐resolution mass spectrometry [21], to extract exact mass of interest and speculate tentative candidates [22], have demonstrated the capability to detect compounds with minimal differences in accurate mass. It enables detailed structural and compositional analysis and useful for identifying proteins and lignin‐like substances [23, 24], yet it provides relative rather than absolute quantification and requires careful interpretation of structural confidence levels. Paired mass distance (PMD) [25, 26] and Kendrick mass defect (KMD) analysis [27] can link transformation of DON molecules. Furthermore, data‐independent acquisition by the thinking of proteomics can capture all detectable peptides in an injection [28] and allows for the identification and quantification of a large number of proteins [29], but peptide detectability remains dependent on digestion efficiency, ionization efficiency, and matrix effects. No single technique is sufficient to resolve the full transformation pathway from macromolecular DON to inorganic nitrogen. Therefore, multi‐level data from protein to AAs need to be integrated to comprehend structure‐dependent reactivity of DON transformation. The application of these complementary approaches, spanning from macromolecules to small monomers, provides a framework for inferring the characteristics of intermediate conversion steps that cannot yet be directly quantified.

To investigate the constraints on nitrogen release from DON, a sequential anaerobic‐microaerobic reactor was adopted under stable operating conditions. The shift from anaerobic to microaerobic conditions was used to assess whether altered redox potential could promote nitrogen release. Multilevel data from macromolecules to monomers of DON (i.e., proteins, peptides, AAs, purines) were adopted to resolve correlations between structural‐dependent characteristics of DON molecules and nitrogen release efficiency. This study identifies a fundamental structural barrier in the stepwise transformation of organic to inorganic nitrogen under varying redox, with implication for understanding the persistence of DON in the natural and engineered systems.

## 2 Materials and Methods

### 2.1 Experimental Setup and Operation

**FIGURE 1:**
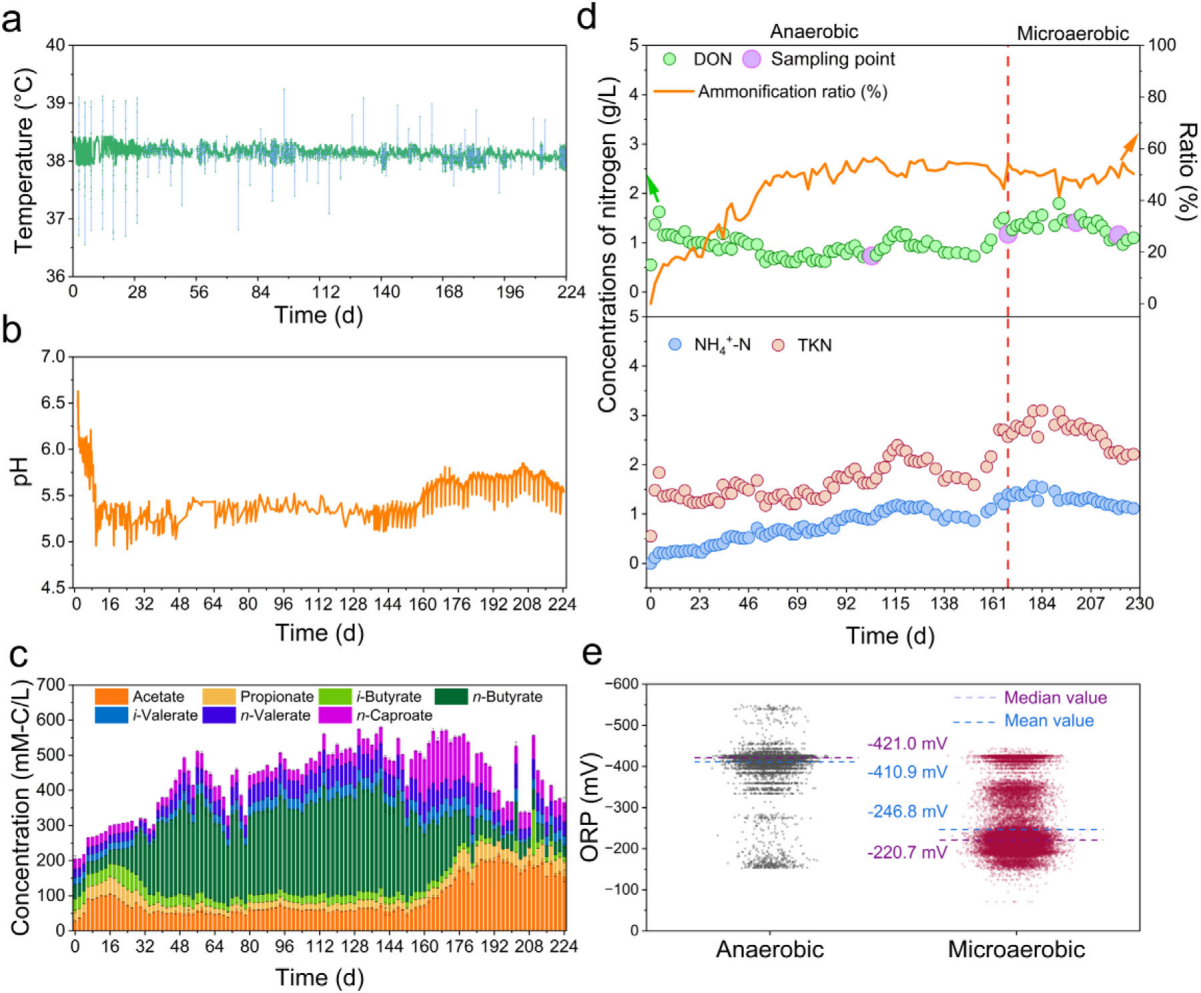
Physiochemical reactor performance of the DON transformation. (a) Real time temperature monitoring curve. (b) pH variation curve. (c) Concentration of VAFs. (d) Concentration of nitrogen. (e) The distribution of varying ORP.

A 5 L reactor with a working volume of 3 L and a headspace volume of 2 L was used in this study, with the headspace designed to buffer gas accumulation during fermentation. The reactor was equipped with an online monitoring (Text S1), and a schematic diagram was provided in Figure S1. Soybean slurry was selected as the substrate given its high content of proteins, peptides, AAs, purines, and other nitrogenous organic compounds, making it a representative DON source. Temperature fluctuations of 1.5 C–1.8°C associated with substrate feeding recovered to the set point within 30 min, representing only ≈0.4% of the total operation time in Figure 1a. Oxidation‐reduction potential (ORP) served as the primary control parameter for shifting reactor conditions from anaerobic (days 0 – 168) to microaerobic operation (days 169 – 224) by continuously supplying filtered air at a controlled flow rate. ORP set points for each phase were summarized in Table S1.

### 2.2 Basic Physicochemical Properties

Basic physicochemical properties were measured on liquid samples filtered through 0.45 µm membrane (Whatman, the UK), including dissolved organic carbon (DOC), dissolved nitrogen (DN), ${\mathrm{NH}}_{4}^{+}-N$, ${\mathrm{NO}}_{3}^{-}-N$, TKN, dissolved short‐chain carboxylates and alcohols (C_2_‐C_6_). Gas‐phase composition (CO_2_, CH_4_, and H_2_) was monitored in the headspace of the fermentation reactor. Analytical methods were described in Text S2. ORP, pH, and temperature were recorded continuously by online sensors. DON was determined by Equation (1).

(1) $$\mathrm{DON}=\mathrm{TKN}-{\mathrm{NH}}_{4}^{+}-N$$

### 2.3 Targeted Analysis of AAs and Purines

All reagents were listed in Text S3. Samples were randomly selected and reconstituted as described in Text S4,S5, following non‐derivative pretreatment procedures adapted from established methods [30, 31, 32]. Chromatographic separation was performed on a BEH Amide column (2.1× 150 mm, 2.5 µm, Waters, USA) as described in Text S6. Separated AAs and purine derivatives were detected by a triple quadrupole mass spectrometer (SCIEX Triple Quad 6500 +, USA) equipped with an electrospray ionization (ESI) source, coupled to a high‐performance liquid chromatography (HPLC) system (Text S6). A total of 38 nitrogen‐containing standard compounds, including 18 amino acids and 20 purine/pyrimidine‐related compounds, were initially screened for targeted analysis. Following analytical method evaluation, 27 compounds (18 amino acids and 9 purine/pyrimidine‐related compounds) met the criteria for reliable quantification and were therefore selected for reactor sample analysis. Detailed preparation and analytical procedures were provided in Text S4. Ion information of the standards were listed in Tables S2,S3, and standard calibration curves for amino acids were shown in Figure S2. Limits of detection and quantification for target analytes were reported in Table S4 and Text S7.

### 2.4 Nontargeted HRMS Analysis of DON and Data Mining

DON was extracted in triplicate via solid phase extraction (SPE) using manually filled mixed‐sorbent cartridges (Mix‐SPE) and Bond Elut PPL cartridges (100 mg, 3 mL, Agilent) (PPL‐SPE) according to methods modified from Li et al. [33]. Detailed SPE and sample analysis procedures were provided in Texts S7,S8. Given that negative ion mode was particularly suited for the identification of DON [34], the transformation of DON was primarily evaluated from mass spectra obtained in ESI (‐) mode with ESI (+) mode for complementary confirmation.

All samples and procedural blank were tested in triplicate, and DON molecules were only recognized if they were retrieved in at least two parallel aliquots. Molecules present in blanks were deducted to obtain the final DON dataset. Tentatively identified compounds were cross‐referenced against *ChemSpider* and *Pubchem* database (Text S9). Molecular characteristics (retention time (RT) and mass‐to‐charge ratio (m/z)) and Venn diagrams were combined to categorize DON. Preprocessed masses from all samples and corresponding identified compounds from Compound Discoverer 3.2 (Thermo Fisher Scientific, Inc., USA) were assigned as transformed, persisted, and produced according to Equation (2) [35, 36].

(2) $$\mathrm{mass}\;\mathrm{type}=\left\{\begin{array}{c} A_{i}>1.3\cdot A_{i+1},\mathrm{transformed}\\ A_{i}<0.7\cdot A_{i+1},\mathrm{produced}\\ 0.7\cdot A_{i+1}<A_{i}<1.3\cdot A_{i+1},\mathrm{persisted} \end{array}\right.$$

where A_i_ and A_i + 1_ indicate the peak area of a given mass feature in consecutively collected samples.

Double bond equivalence and aromaticity index (AI_mod_) were calculated following Koch and Dittmar [37] to assess the recalcitrance of identified compound. Peak areas across duplicate samples were processed to retain only mass features and molecular formulas consistently detected across replicates. Molecular transformation was inferred by matching PMD to entries in Table S5 thereby constraining the number of candidate formulas. This directed PMD analysis was performed in the RStudio environment (v4.4.1) with the R packages *tidyverse* and *RMThreshold* [35]. PMD pathways connecting “transformed” to “produced” were designated as strong transformation links. Full data processing details were described in Text S10.

### 2.5 Analysis of Proteins and Peptides

Proteins were digested with trypsin for 12 h as described in Text S11. Total concentrations of proteins were summarized in Table S6. Peptides were analyzed by LC‐MS using a Vanquish Neo system coupled to an Orbitrap Astral mass spectrometer (Thermo Fisher Scientific, USA). Chromatographic separation was carried out on a uPAC High Throughput column (75 µm×5.5 cm, Thermo, USA) with a mobile phase flow rate of 1.25‐2.5 µL/min. MS/MS spectra were acquired in data‐independent acquisition mode as described in Text S11. The persistence of proteins, peptides, and AAs across all samples was assessed according to Equation (3). The category principles of proteins and peptides were described in Text S11.

(3) $$Q_{s}=\frac{\mathrm{Valu}e_{\mathrm{anaerobic}}}{\mathrm{Valu}e_{\mathrm{microaerobic}}}$$

where *S* refers to proteins, peptides, formulas of DON or AAs, and *Value* refers to the signal intensity of a given protein or peptide, or the measured concentration of an AA. Categorization of proteins and peptides followed the same principles as Equation (2) and was further described in Text S11.

The nitrogen contribution of proteins and the nitrogen contribution intensity of peptides were calculated by Equations (4) and (5), respectively.

(4) $$N\;\mathrm{contribution}=\mathrm{lo}g_{10}\left(\mathrm{mol}\;N/\mathrm{mol}\;\mathrm{peptide}\times \mathrm{intensity}\right)$$

(5) $$\begin{array}{ccc} N\;\mathrm{contribution}\;\mathrm{intensity} & = & \log_{10}\;(\left(\mathrm{relative}\;\mathrm{quantitation}\right)\\ & & \times \;(\mathrm{mol}\;N/\mathrm{mol}\;\mathrm{peptide})) \end{array}$$

Proteins and peptides were compared between anaerobic and microaerobic groups by T‐test, with *p*‐values calculated from per‐protein or per‐peptide intensity distributions. Molecular weight (MW)‐dependent significance was also assessed by T‐test across MW bins. Peptides exhibited no significant differences across all samples served as the reference level. Multinomial logistic regression (R package *nnet*, RStudio v2025.9.2.418) was subsequently applied to the relative amino acid composition of classified peptide groups to identify compositional features associated with persistence, independent of absolute quantitative values.

A nonapeptide model (REGDMFVWL, C_53_H_77_N_13_O_14_S_1_) was by integrating the amino acid composition of the soybean substrate, the multinomial logistic regression coefficient matrix, and the nitrogen contribution values of individual amino acids. Two additional model peptides (DWSFILK, C_45_H_66_N_9_O_11_, and DELDIVIPTIR, C_57_H_100_N_14_O_19_) were selected directly from the LC‐MS/MS‐identified peptide dataset based on the logistic regression coefficient matrix. Secondary structure prediction was performed using PEP‐FOLD 4 [38]. Geometry optimization and vibrational frequency calculations were conducted at the B3LYP‐D3BJ level of theory using Gaussian 16 [39]. FMO energies and Fukui descriptors were computed using Multiwfn (v.3.8) [40, 41]. Molecular visualizations were generated in VESTA [42]. The rationale for selection of AAs and full computational procedures was provided in Text S12, and isosurface representations of the electronic properties for all three peptide models were shown in Figures S3,S4.

## 3 Results

### 3.1 Basic Physiochemical Parameters During DON Transformation

The organic loading rate increased gradually from day 0 to day 4. Over the subsequent 220 days, concentrations of COD and DOC were 24 to 40 g COD/L and 6.6 to 11.1 g C/L, respectively, covering 90% of the actual value distribution range (Figure S5). Alcohols were not detected at any stage. Temperature of the system was set at 38 ± 0.3°C (Figure 1a). Accumulation of short‐chain fatty acids drove a decline in pH [43]. pH fluctuated between 5.11 and 5.50 during the middle anaerobic stage (days 32–156) (Figure 1b), with hydrolysis and acidification proceeding unimpaired. When pH fell below 6.0, it inhibited the activity of methanogenic microbes, and CH_4_ was not detected in the headspace of the system (Figure S6). In the later anaerobic period (days 80–156), pH ranged from 5.20 to 5.50, and increased to 5.30–5.85 in microaerobic stage (days 168–224). The gradual upward shift might be attributed to the progressive stabilization of volatile fatty acid production and the modest buffering effect of continued ${\mathrm{NH}}_{4}^{+}$ accumulation. During days 0–28, concentrations of acetate, propionate, *i*‐butyrate, *n*‐butyrate, *i*‐valerate, *n*‐valerate, and *n*‐caproate were 152 ± 52, 76 ± 19, 81 ± 21, 86 ± 23, 78 ± 20, 86 ± 21, and 104 ± 28 mmol C/L, respectively (Figure 1c). H_2_ in the headspace raised up to 8% on day 112 (Figure S6). During days 158–168, the concentrations of acetate and *n*‐caproate increased from 69 ± 2 mmol C/L and 120 ± 5 mmol C/L to 120 ± 2 mmol C/L and 149 ± 57 mmol C/L, respectively, while *n*‐butyrate increased from 220 ± 20 mmol C/L to 310 ± 20 mmol C/L during the stable anaerobic stage (days 80–156). In microaerobic stage (from day 168 on), H_2_ decreased to undetectable levels. The selectivity of *n*‐butyrate decreased from 53% ± 2% (days 80–156, 270 ± 30 mmol C/L) to 11% ± 3% (days 182–224, 47 ± 18 mmol C/L), while that of acetate increased from 13% ± 2% (days 80–156, 61 ± 6 mmol C/L) to 46% ± 8% (days 182–224, 190 ± 20 mmol C/L) over the same period.

After a 24‐day operation (3 hydraulic retention times, HRT), the concentration of ${\mathrm{NH}}_{4}^{+}-N$ increased threefold compared to day 4, with the ${\mathrm{NH}}_{4}^{+}-N$/TKN (total Kjeldahl nitrogen) ratio reaching 27.5% (Figure 1d). After 10 HRT (days 80–156), the concentrations of TKN and ${\mathrm{NH}}_{4}^{+}-N$ were 1.85 ± 0.27 g‐N/L and 0.98 ± 0.14 g‐N/L, respectively. Despite variations in the composition and concentration of VFAs, the concentration of ${\mathrm{NH}}_{4}^{+}-N$ increased from 0.89 ± 0.02 g/L (day 80) to 1.31 ± 0.02 g/L on day 164, whereas the ratio of ${\mathrm{NH}}_{4}^{+}-N$/TKN remained stable (52% ± 3%), indicating that there composition of VFAs had no significant bearing on the ammonification rate of DON. During the microaerobic stage, the concentrations of ${\mathrm{NH}}_{4}^{+}-N$ and TN increased to 1.33 ± 0.13 g‐N/L and 2.68 ± 0.27 g‐N/L, respectively, with DON/TN remained at 50% ± 3%. Thereby, the stable performance of ammonification across both redox stages provided a controlled basis for resolving the persistence of DON from protein to AA level.

### 3.2 The Persistence of DON at Protein‐Level

**FIGURE 2:**
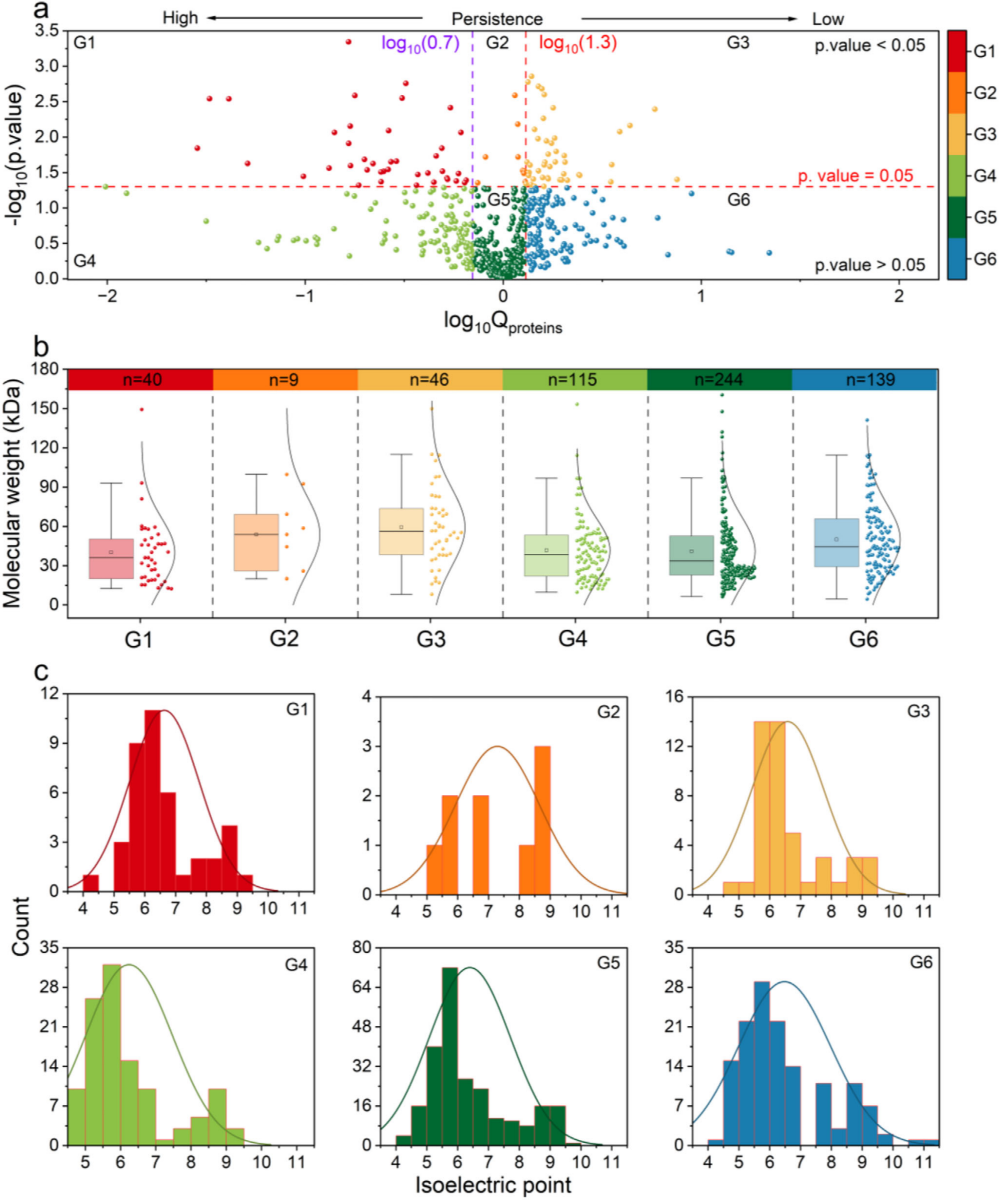
Persistent of DON at protein‐level. (a) Volcano diagram for the persistence of DON at different protein ‐level. (b) MW distribution of different groups of proteins. (c) Isoelectric point distribution of recognized proteins. Classification principle: G1: p.value < 0.05, Q_proteins_ ≥ 0.7; G2: p.value < 0.05, 0.7 < Q_proteins_ ≤ 1.3; G3. p.value < 0.05, Q_proteins_ ≥ 1.3; G4: p.value > 0.05, Q_proteins_ ≤ 0.7; G5: p.value > 0.05, 0.7 < Q_proteins_ ≤ 1.3; G6: p.value > 0.05, Q_proteins_ ≥ 1.3. Q_proteins_ ≤ 0.7 represents degraded proteins in anaerobic system; Q_proteins_ > 1.3 represents persistent proteins in micro‐aerobic system; 0.3 < Q_proteins_ ≤ 1.3 represents proteins stable in the system.

A total of 668 proteins were identified, with MW ranging from 4.5 to 200 kDa. Among these, 63 uncharacterized proteins were attributed to denaturation products of proteins under acidic pH, and six proteins were detected exclusively in the anaerobic stage. Of the remaining 593 proteins detected across all samples (Figure 2a), six persistent levels were defined based on Q_proteins_ (Equation 3). Therefore, persistence of DON at protein level followed the order G1 ≈ G4> G2 ≈ G5> G3 ≈ G6, with G1 and G4 representing the most persistent fraction and G3 and G6 containing proteins most susceptible to transformation under microaerobic stage.

Approximately 34.9% of proteins had MW exceeding 50 kDa (Figure 2b). Proteins in G3 and G6 were disproportionately large, with 45% exceeding 50 kDa. The MW distributions of G4 and G5 differed significantly (p.value = 0.01, two‐tailed T‐test), consistent with microaerobic conditions promoting hydrolysis of residual solid‐phase proteins [44]. G5 was notably enriched in high‐MW proteins, with 42.5% having a MW of 160.4 kDa. The distribution of isoelectric points (Figure 2c) indicated that 56% of proteins would carry a positive charge under the operating ORP range of ‐400 mV to ‐100 mV (Figure 1e), suggesting that positively charged AAs contribute more to the overall charge profile than negatively charged ones. in contrast, the more persistent proteins in G1 and G4 were enriched in negatively charged residues. Moreover, over 85% of protein categories were identified in both redox stages, indicating that the bulk protein composition remained relatively stable throughout operation. This stability provided a foundation for subsequent investigation of DON transformation reactivity at the levels of molecular formula, peptide, and AA.

### 3.3 Molecular Characteristics and Transformation of DON

A total of 112 persistent DOM molecules were identified in samples by PPL‐SPE, compared to 237 in samples by Mix‐SPE (Figure S7). In both cases, molecules in the 200–500 Da range dominated the persistent DON pool, contributing to 86.5% and 89.6% of the total by PPL‐SPE and Mix‐SPE, respectively. In PPL‐SPE samples, DON formulas comprised 61% and 73% of all detected formulas in anaerobic and microaerobic stages, respectively, yet persistent DON formulas accounted for only one‐third of the total (Figure S8). This indicates that a substantial fraction of DON was retained in organic forms rather than fully mineralized, and that transformations of both non‐N‐containing and N‐containing functional groups occurred concurrently.

**FIGURE 3:**
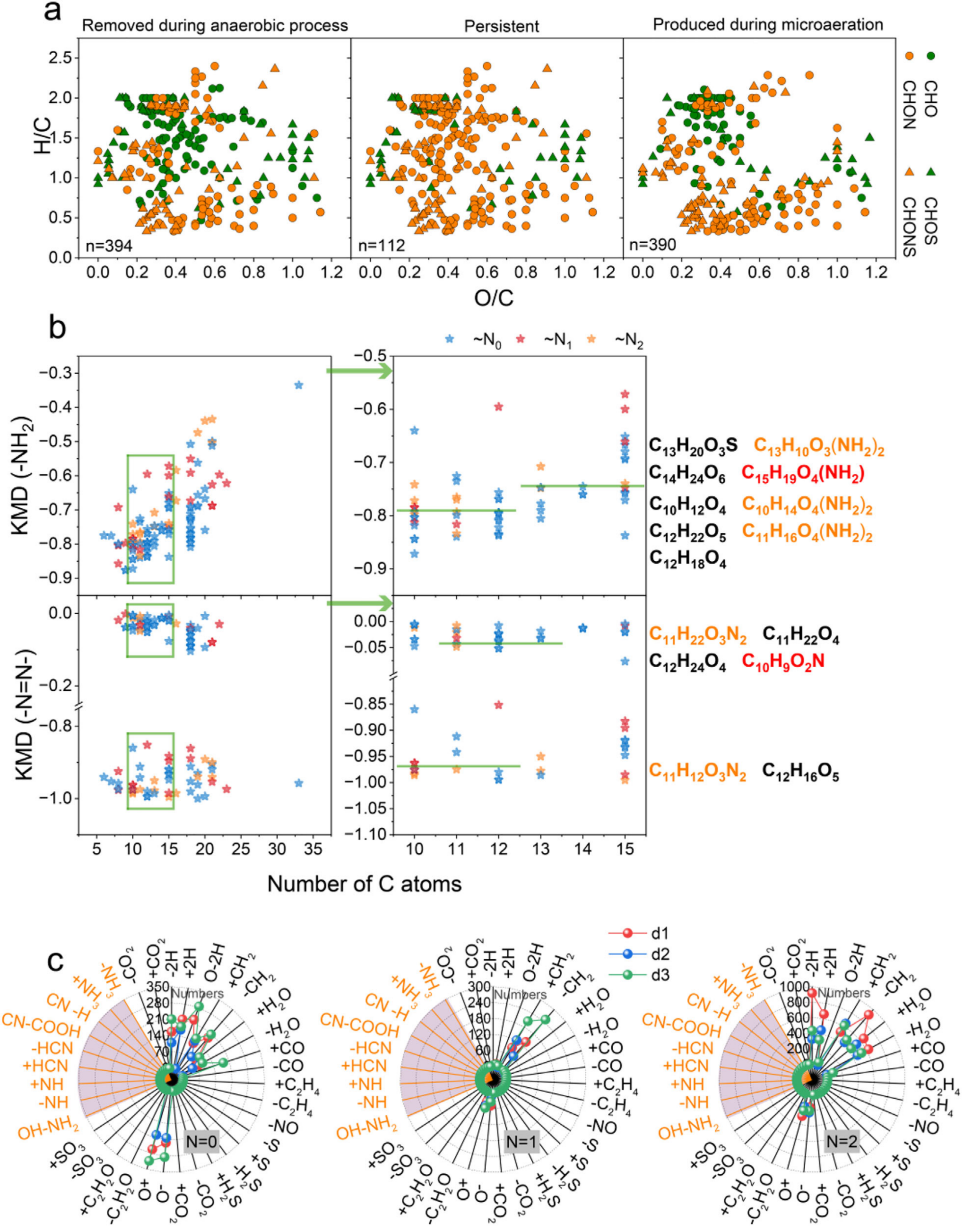
Molecular characteristics and potential transformation pathways of DON at the level of molecular formula obtained under ESI (−) acquisition mode using PPL‐SPE pretreatment. (a) Van Krevelen Diagram of molecular formulas. (b) KMD of persistent DON recognized in the system. (c) The distribution of transformation paired pathways. (d1): Paired two anaerobic samples; (d2): Paired anaerobic and microaerobic samples; (d3): Paired two microaerobic samples.

The number of unique formulas identified under anaerobic stage was approximately 1.6‐fold greater than under microaerobic stage, suggesting that elevated ORP promoted the transformation of DON. However, oxygen injection increased the weighted average H/C ratio of the bulk DOM pool in ESI (‐) mode to above 1.7 (Figure 3a), indicating a shift toward more saturated or aliphatic structures [45]. The weighted average N/C ratio of bulk DOM decreased following oxygen injection, while that of the persistent DON pool remained largely unchanged. DON molecules with more oxygen atoms tend to carry more carboxylic and carbonyl groups, contributing to greater double bond equivalence values and structural complexity (Figure S9) [46]. Constant with this, an increase of oxygen content in DON [47] following oxygen injection after day 168 was reflected in the KMD distribution of persistent DON (Figure 3b), with oxygen atom number influencing molecular structural features [48]. Taken together, these observations indicate that anaerobic stage supported the accumulation of DON molecules, whereas microaeration had limited effect on the ammonification of persistent DON.

To identify the critical limitations, the relative abundance of persistent DON molecules was compared across samples (Figures S10,S11), revealing a consistent distributional profile throughout the incubation. PMD analysis was subsequently performed on all detected molecular formulas (Figure S11), with Figure 3c summarizing the number of nitrogen‐containing PMD pairs identified in PPL‐SPE samples. Ten reaction categories dominated the paired pathways, including ±15.9949 (± O), ± 2.0156 (± 2H), ±14.0156 (± CH_2_), ±18.0106 (± H_2_O), +13.9793 (O‐2H), +27.9950 (+CO) [49]. DOM containing two nitrogen atoms accounted for 71.4% of paired pathways in d1, approximately 0.32% higher than that in d2 and 15% higher than that in d3, respectively. The number of paired pathways for DOM containing two nitrogen atoms decreased by 31.8% in d2, while the total pathways in d3 were 1.3‐fold those in d2, suggesting that DOM containing two nitrogen atoms exhibited greater structural reactivity under anaerobic than microaerobic stage. Typically, demethylation (‐ CH_2_) was the predominant pathway in d1, whereas methylation (+ CH_2_) dominated in d2 and d3. For DOM containing no nitrogen, + O (16.6%) and—O (15.2%) were two most abundant pathways in d1‐d3, while + CO (4.0%) was the least frequent, occurring exclusively in d3. Demethylation accounted for 38.7% of pathways for DOM containing one N atom across samples, whereas methylation accounted for 35.6% of pathways involving DON containing two N atoms. As a complementary result, there were 12 kinds of pathways occurring in samples by Mix‐SPE (Figure S12), and over 60% of pathways were unrelated to transformation of DON. DON‐related PMD accounted for 72.9% in PPL‐SPE samples and 37.3% in Mix‐SPE samples, with strong PMD reaching 13.6% and 14.3%, respectively. Notably, although nitrogen‐involving PMD pathways (e.g., ±NH_3_, ±HCN, −CN+COOH) were explicitly included in the library, none were matched across d1‐d3, indicating that corresponding nitrogen‐releasing molecular transformation relationships were not resolved within the detected dissolved organic molecular features under the present analytical framework.

KMD (‐N = N‐) clusters were predominantly distributed in the range of ‐0.2 to 0 Da and ‐1.0 to ‐0.8 Da, with few persistent DON molecules identified in these clusters. The absence of ‐NH_2_ PMD among formulas below 1000 Da is consistent with the lack of KMD(‐NH_2_) signals in Figure 3b, collectively suggesting that all the assigned N‐containing compounds underwent transformation primarily through pathways not involving N atoms. Given that the detected DON originated from hydrolysis of macromolecules, these findings indicate that the limitation for the ammonification of macromolecular DON does not lie in the hydrolysis step itself.

### 3.4 Structural Dependent Nitrogen Contribution Intensity of Peptide

**FIGURE 4:**
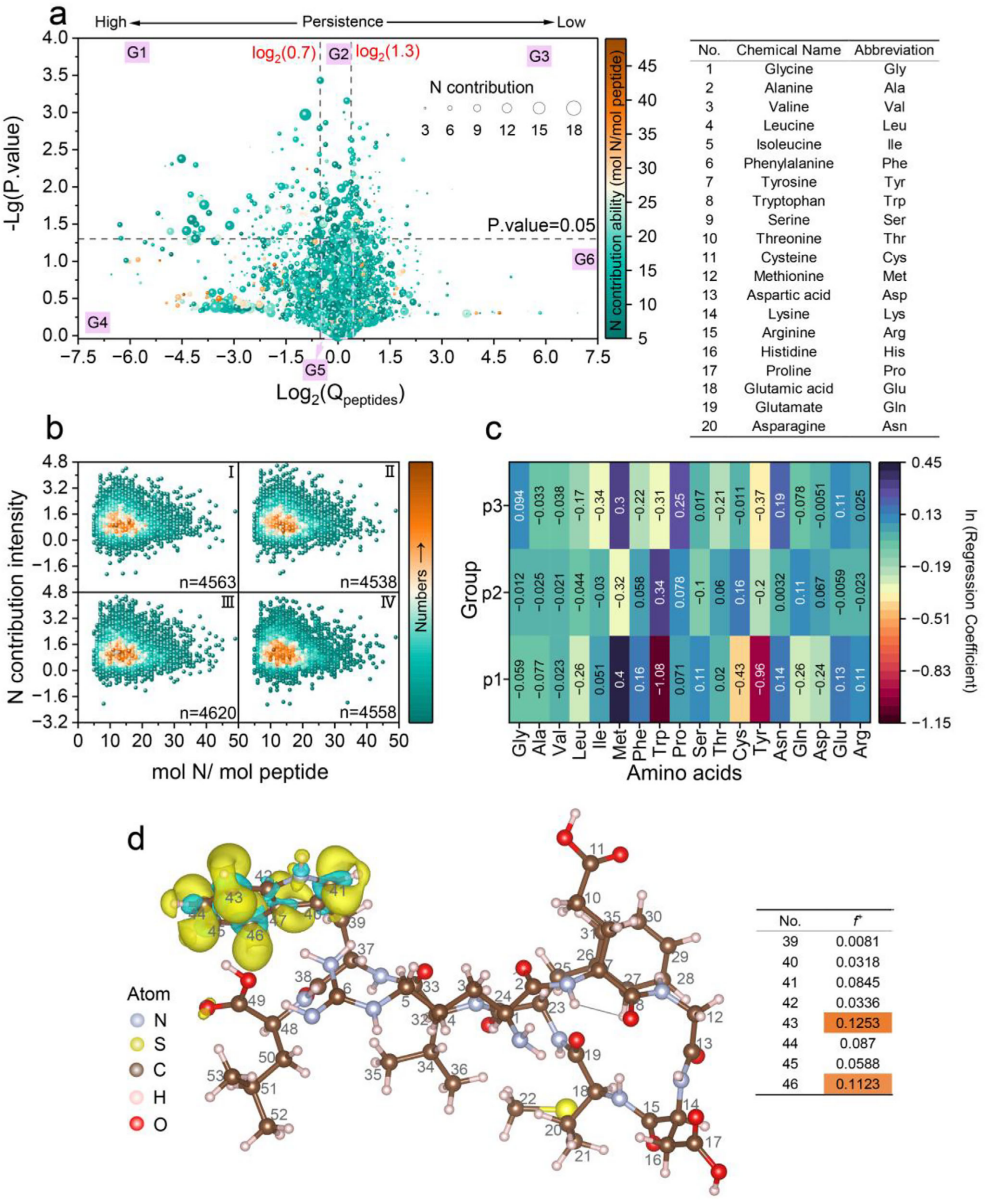
DON persistence at the level of peptide. (a) Volcano diagram of recognized persistent peptides with N contribution intensity. (b) N contribution intensity of peptides by multinomial logistic regression analysis. (c) The degree of associations between peptides and its basic units. (d) Fukui *f^+^* function map of nonapeptide. Classification principle: G1: p.value < 0.05, Q_peptides_ ≥ 0.7; G2: p.value < 0.05, 0.7 < Q_peptides_ ≤ 1.3; G3. p.value < 0.05, Q_peptides_ ≥ 1.3; G4: p.value > 0.05, Q_peptides_ ≤ 0.7; G5: p.value > 0.05, 0.7< Q_peptides_ ≤ 1.3; G6: p.value > 0.05, Q_peptides_ ≥ 1.3. Q_peptides_ ≤ 0.7 represents degraded peptides in anaerobic system; Q_peptides_ > 1.3 represents persistent peptides in micro‐aerobic system; 0.3 < Q_peptides_ ≤ 1.3 represents peptides stable in the system. p1: Results of G1. p2: Results of G2. p3: Results of G3. The f+ values indicate the regions susceptible to nucleophilic attack.

Peptides, as the typical components from the hydrolysis of proteins, were represented by 4,780 species identified with MW of 400–5500 Da, of which 4,079 were detected across all samples. Peptides were also divided into six groups (Figure 4a) according to the Eq.3, with sample counts of 4,563, 4,538, 4,620, and 4,558 across the four sampling points shown in Figure 1e. Figure 4b illustrated the theoretical N contribution value per peptide (mol N/mol peptide). Of these, peptides in G1, G2, and G3 demonstrated significant differences of intensity in anaerobic and microaerobic stages. Most of peptides had theoretical N contributions of 8–20 mol N/mol peptide (Figure 4b), likely reflecting the low abundance of Arginine (Arg, N contribution = 0.32 mol N/mol) within these peptides.

To investigate the structural determinants of peptide persistence, multinomial logistic regression was applied using the relative abundance of 20 AAs as predictors, with peptides in G5 as reference baseline (Figure 4c). Methionine (Met) carried regression coefficients of e^0.40^ and e^0.3^ in p1 and p3, respectively, but e^−0.32^ in p2. This suggested Met as a structural indicator of redox‐dependent stability, and peptides containing more Met showed a greater tendency to be transformed under fluctuating ORP. The regression coefficient of asparagine (Asn) was 0 in p2, indicating no contribution to the persistence of peptides under those conditions. Although regression coefficients of tryptophan (Trp) were e^−1.08^ in p1 and e^−0.31^ in p3, respectively, the largest regression coefficient of e^0.34^ in p2 suggested that Trp‐containing peptides were preferentially retained in anaerobic stage and selectively degraded under anaerobic stage. Proline (Pro) exhibited a notably high regression coefficient of e^0.25^ in p3, exceeding its contributions in other groups. These results showed that Met and Trp emerged as the primary structural determinants of peptide persistence across redox conditions. Notably, both residues ranked among the least abundant amino acids in the full detected peptide pool (Met: 19^th^, 1.4%; Trp: 20^th^, 1.2%; Figure S13), confirming that their association with persistence reflects a compositional feature of the stable fraction rather than a detection artefact.

Three model peptides (AILNMFGMK, DWSFILK, and DELDIVIPTIR) were used for FMO and Fukui function analysis, as described in the Methods section. The nonapeptide yielded a HOMO‐LUMO gap of 7.06 eV, indicating relatively low global electron‐transfer propensity [50]. The supplementary peptides showed HOMO‐LUMO gaps of 5.14 eV (DWSFILK) and 5.93 eV (DELDIVIPTIR), suggesting broadly comparable electronic stability across peptides of differing AA compositions. To further resolve site‐specific reactivity of medium MW nitrogenous intermediates, Fukui indices were calculated to map potentially associated with electrophilic, nucleophilic, and radical interactions [51]. For the reference peptide, elevated values were confined to a limited number of carbon positions (Figure 4d, Table S7), including C43 (0.1253) and C46 (0.1123) for *f^+^*, and C40 (0.1311), C41 (0.1154) for *f^−^*, and C41 as the highest radical‐associated position (*f^0^* = 0.1) during the microaerobic stage. Similar localization patterns were observed in DWSFILK and DELDIVIPTIR, where elevated Fukui indices were primarily restricted to side‐chain positions or terminal atoms rather than backbone amide bonds, with the Trp indole ring carbons dominating local reactivity in DWSFILK and terminal nitrogen atoms accounting for the highest *f^−^* values in DELDIVIPTIR (Figure S4, Tables S8,S9). Taken together, these computational results provide a hypothesis‐generating perspective on potential differences in local molecular reactivity among peptide structures. The predominance of elevated Fukui indices outside backbone amide bonds is consistent with the possibility that some soluble medium‐MW nitrogenous compounds may resist nonspecific transformation under weakly acidic fermentation conditions, though this should not be interpreted as a mechanistic explanation of peptide persistence across the detected pool.

### 3.5 The Persistence of AAs and Purines

**FIGURE 5:**
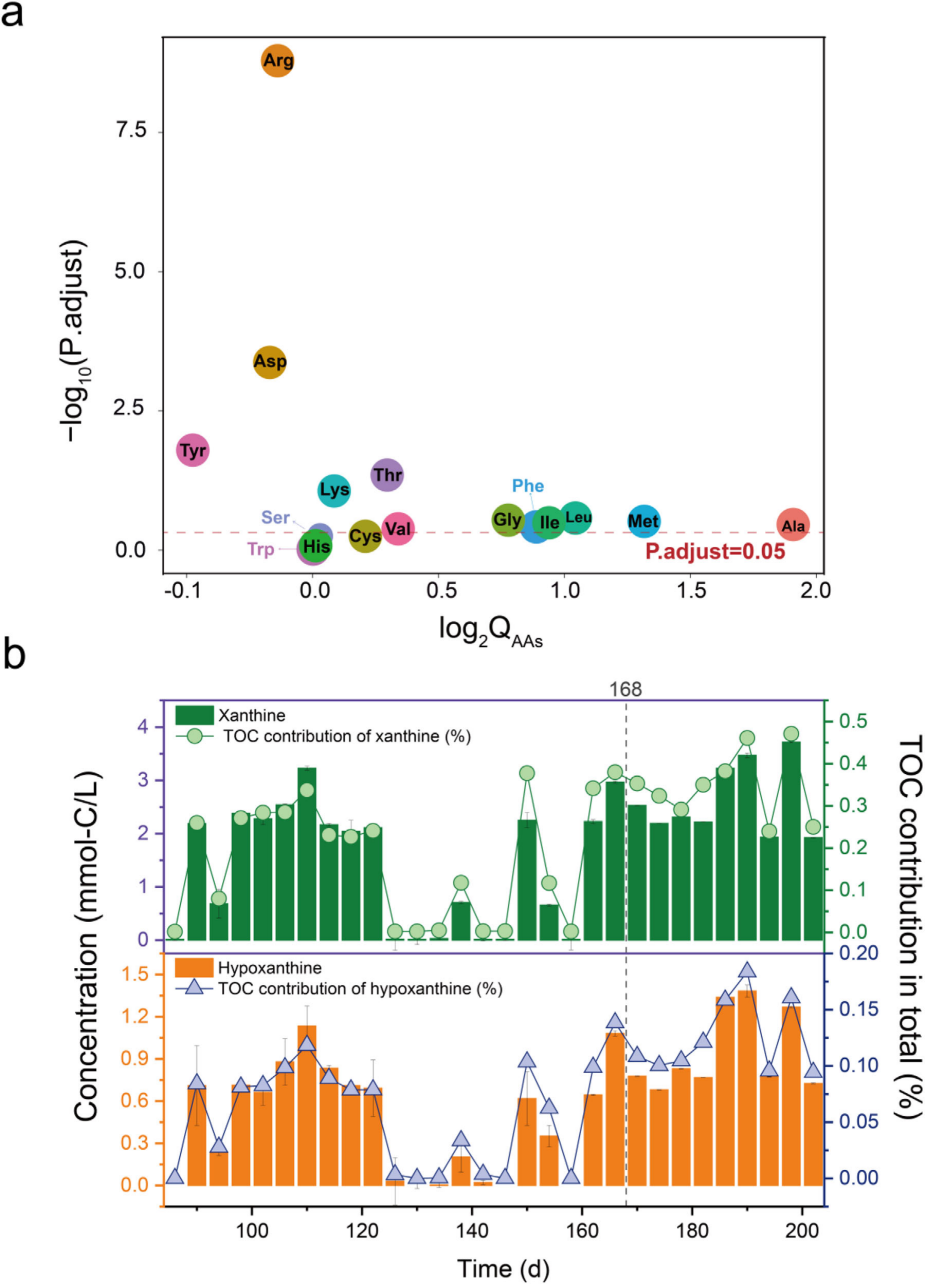
The concentrations of AAs, xanthine and hypoxanthine in collected samples. (a) Volcano diagram of 13 kinds of AAs. (b) Concentrations of xanthine and hypoxanthine.

Among all detected AAs, Q_AAs_ of Tyr was the lowest (Figure 5a), though the average concentration gap was only 0.34 mmol C/L. The average concentration of arginine (Arg) and aspartic acid (Asp) ranged from 0.13 to 0.21 mmol C/L and 0.25 to 0.59 mmol C/L over days 28–222, respectively. Asp had an extra carboxylic group in its side chains, providing two potential sites of deprotonation [52] and contributing to its readily reactivity. Although Asp was among the most abundant AAs in soybean proteins [53], its concentration increased relative to other AAs following oxygen injection (Figure S14). Cysteine and Met, both sulfur‐containing AAs, were present at relatively low concentrations, consistent with their susceptibility to oxidative degradation. Glutamate could be metabolized to organic acids such as lactate and succinate when oxygen is limited [54], which may account for its behavior during the anaerobic stage. At the current operating pH, most AAs carried a negative charge.

Hypoxanthine was measured over days 86–166, reaching an average concentration of 0.45 ± 0.39 mmol C/L and falling below the detection limit by Day 130 (Figure 5b). In the microaerobic stage, the concentrations of xanthine and hypoxanthine (2.7 ± 0.7 mmol C/L and 0.95 ± 0.29 mmol C/L) were significantly higher than those in the anaerobic stage (1.36 ± 0.45 mmol C /L and 1.17 ± 0.39 mmol C /L). Both compounds are intermediate products in the degradation of purine nucleotides, with hypoxanthine oxidized to xanthine by xanthine dehydrogenase (XDH) and xanthine subsequently converted to uric acid [55].

Figure 5a shows that 16 kinds AAs were quantified, and 13 kinds of AAs were degraded and maintained a low concentration (1.13–1.80 mmol C/L) in microaerobic stage, with total N contribution to TKN below 2%. Of the 26 N‐containing standards selected for the analysis of purines and its metabolites, only xanthine and hypoxanthine showed apparent intensity exceeding three times the signal‐to‐noise ratio across all samples (Figure 5b). The TOC contributions of xanthine and hypoxanthine were both below 1%, and their N contribution to TKN was less than 0.15% in total. Therefore, these results indicate that AAs and purines do not constitute the persistent DON in ammonification process, and that the barrier to ammonification resides primarily in the structural stability of medium‐MW nitrogenous intermediates rather than in the small‐molecule pool.

## 4 Discussion

The transformation of DON covers proteins, peptides, pyrimidines, and purine‐derivatives, yet stepwise kinetic analysis at environmentally relevant concentrations remains methodologically intractable. Therefore, we designed a steady‐state ammonification reactor to track the distribution of these chemical constituents under varying ORP conditions, and integrating known transformation pathways as an alternative strategy to infer the characteristics of the intermediate conversion steps that cannot yet be directly quantified. Sequential analysis from macromolecules (protein‐level) to constituent units (AAs) bridges the gap between the transformation of DON from macromolecules and the reaction converting DON into inorganic nitrogen. This analysis challenges the conventional assumption that hydrolysis is the primary limitation during anaerobic processing of organic nitrogen. Instead, our findings indicate that the conversion of medium‐MW nitrogenous intermediates represent a key limitation of ammonification, particularly in the conversion of multimeric compounds to bioavailable monomers. A compositional analysis of quantifiable DON fractions at representative time points confirmed this pattern, with dissolved protein‐N accounting for 63%–71% of DON while free amino acid‐N and purine‐N together contributed less than 6%, leaving the remainder attributable to the unquantified peptide fraction (Table S10).

In anaerobic and microaerobic stages, DON molecules could undergo transformation to produce inorganic molecules for biosynthesis, or metabolized to support microbial energy production. More than 85% of detected proteins were consistently present across all sampling points and matched predominantly against a soybean protein database, with samples collected immediately prior to each feeding event. This temporal stability across both anaerobic and microaerobic phases, combined with the dissolved‐phase nature of all detected proteins (0.45 µm filtered), suggests that solubilization have already occurred and that the downstream conversion of these dissolved intermediates represents the more constrained step. This pattern is further reflected by PMD analysis, where nitrogen‐involving pathways such as ±  NH_3_, ± HCN, and—CN + COOH were included in the matching library but returned no matches across d1‐d3, while nitrogen‐containing compounds were largely concentrated in the 200–500 Da range. Although PMD analysis characterizes potential structural relationships among detected molecules rather than directly demonstrating net ammonification, the stable ${\mathrm{NH}}_{4}^{+}/\mathrm{TKN}$ ratio (52% ± 3%) throughout the anaerobic phase suggests that the proportion of nitrogen mineralized remained broadly constant over time. While this observation does not directly validate PMD‐inferred transformation relationships, it is consistent with the interpretation that persistent DON may be associated with limited downstream nitrogen release. Free AAs and purine‐derivatives remained minor contributors to the DON pool throughout, suggesting relatively rapid turnover at the monomeric stage.

The findings of this study are most directly applicable to DON pools dominated by primary‐source proteinaceous nitrogen, such as those encountered in food waste fermentation and municipal wastewater influent. These results indicate that improving ammonification efficiency in such systems would benefit more from strategies targeting the release of monomeric nitrogen from peptide‐bound intermediates than from further enhancement of macromolecular hydrolysis, which does not appear to be the bottleneck under the conditions examined. However, DON in natural aquatic environments and engineered treatment systems is highly heterogeneous, encompassing not only primary‐source proteins but also microbially derived fractions [56], including extracellular polymeric substances, cell lysis products, and secondary metabolites generated during processes such as anaerobic digestion of excess sludge. These secondary‐source DON fractions differ substantially in molecular structure and degree of prior microbial processing, and the conclusions drawn here should not be extended to such systems without independent verification. Nevertheless, the multi‐level analytical framework developed in this study, integrating protein‐level persistence analysis, nontargeted HRMS formula tracking, peptide‐level proteomics, and targeted monomer quantification, provides a transferable approach for characterizing DON transformation across systems of varying substrate origins. Applying this framework to secondary‐source DON pools, including sludge digestates, sedimentary organic matter, and algal bloom‐derived nitrogen, would enable systematic cross‐system comparison and clarify the extent to which the peptide‐nitrogen bottleneck generalizes across DON source types.

Our findings reinforce the view that hydrolysis is the primary limitation in traditional wastewater and organic waste treatment, where enhancing hydrolysis is a common technique employed to accelerate the degradation of DON. The present results indicate, however, that hydrolysis is not the limiting step under the conditions examined, and that the conversion of soluble medium‐MW nitrogenous intermediates to bioavailable monomers represents a more critical and underexploited target. The nonapeptide modelling further suggests a structural basis for this resistance. Met was identified as an indicator of peptide stability across ORP conditions, and Trp appeared particularly associated with peptide persistence under anaerobic conditions, together pointing to specific AAs in peptide as potential determinants of resistance to further degradation. The enrichment of hydrophobic, oxidation‐resistant residues such as Met and Trp in persistent peptides, identified here through multi‐level mass spectrometric and statistical analyses, provides a molecular mechanism consistent with selective AA preservation reported in both engineered and natural diagenetic environments. Although the specific MW range and AA composition of recalcitrant fractions will vary with substrate origin and microbial community composition, the principle that peptide‐bound nitrogen in certain structural configurations constitutes limitation for nitrogen mineralization is expected to apply broadly to systems receiving primary‐source proteinaceous inputs.

From an engineering perspective, future process development for nitrogen‐rich biomass treatment should prioritize strategies that promote the release of monomeric nitrogen from peptide‐bound intermediates, whether through targeted enzymatic augmentation, redox modulation, or physicochemical pretreatment, rather than focusing solely on macromolecular solubilization. Such approaches may improve nitrogen recovery efficiency in anaerobic digestion and biological nutrient removal processes, particularly where DON accumulation represents limits recoverable nitrogen.

## 5 Conclusions

This study provides a molecular‐level characterization of DON transformation during ammonification under varying redox conditions. The results demonstrate that over 85% proteins are persistent across redox variations with Met and Trp identified as typical components of persistent peptides in the system. Further validation using FMO calculations with three established peptide models reveal that ammonification is not limited by protein hydrolysis, but rather by medium molecular weight nitrogenous multimers, identifying a previously overlooked bottleneck in nitrogen mineralization. By linking DON molecular composition to transformation behavior across protein, peptide, and AA levels, this work offers a mechanistic basis for the variability in ammonification efficiency observed across natural and engineered systems, with implications for predicting nitrogen release from organic matter and optimizing nitrogen management under dynamic redox conditions.

## Author Contributions

**P.H**., **F.L**., **H.Z**. and **J.Q**. conceptualized the idea and supervised the project. **J.L**. and **J.Q**. designed the research, carried out experiments, analyzed data, and wrote the manuscript. All authors discussed the results and commented on the manuscript.

## Conflicts of Interest

The authors declare no conflicts of interest.

## Supporting information




**Supporting File**: advs77787‐sup‐0001‐SuppMat.docx.

## Data Availability

The data that support the findings of this study are available from the corresponding author upon reasonable request.
