# Supplementary material for: Ammonification Process Is Limited by Transformation of Medium Molecular Weight Nitrogenous Organics but Not Hydrolysis of Proteins
Source: Adv Sci (Weinh). 2026 Sep 16:e77787. Online ahead of print. doi: 10.1002/advs.77787 (PMC13583228; doi:10.1002/advs.77787)
Supplement: Supplementary file 1 [file ADVS-9999-e77787-s001.docx]

**Supporting Information**

**Ammonification process is limited by transformation of medium molecular weight nitrogenous organics but not hydrolysis of proteins**

Jing Li^1, 2, #^, Junjie Qiu^1, 2, #^, Fan Lü^1, 2, 3^, Hua Zhang^1, 2, 3^, Pinjing He^1, 2, 3, *^

^1^ Institute of Waste Treatment and Reclamation, College of Environmental Science and Engineering, Tongji University, Shanghai, 200092, PR China

^2^ State Key Laboratory of Water Pollution Control and Green Resource Recycling, College of Environmental Science and Engineering, Tongji University, Shanghai, 200092, PR China

^3^ Shanghai Institute of Pollution Control and Ecological Security, Shanghai, 200092, PR China

* Corresponding author: Pinjing He, solidwaste@tongji.edu.cn

**^#^** These authors contributed equally to this work.

**Content**

[Text S1 Experiment set-up and sampling information	1](#_Toc232244521)

[Text S2 Analytical methods for basic physicochemical parameters	1](#_Toc232244522)

[Text S3 Chemicals preparation	2](#_Toc232244523)

[Text S4 Solutions preparation	3](#_Toc232244524)

[Text S5 Samples preparation for targeted analysis	4](#_Toc232244525)

[Text S6 Liquid chromatography mass spectrometry	4](#_Toc232244526)

[Text S7 Solid phase extraction	6](#_Toc232244527)

[Text S8 Operating conditions of Ultra-high Performance Liquid chromatography mass spectrometry	9](#_Toc232244528)

[Text S9 Processing of mass data	10](#_Toc232244529)

[Text S10 Calculations of molecular property indexes	11](#_Toc232244530)

[Text S11 DIA based proteomics	13](#_Toc232244531)

[Text S12 DIA based peptides analysis and model selection	15](#_Toc232244532)

[Supplementary of Figure List	18](#_Toc232244533)

[Supplementary of Table List	33](#_Toc232244534)

[Reference	56](#_Toc232244535)

### Text S1 Experiment set-up and sampling information

There is no adjustment inside the reactor except 800 mL mixture were taken out, and 800 mL of unfermented materials (60g soybean, with a content of 85%) mixing with centrifuged solid residue from 300 mL fermented mixture were feed in for every two days to differentiate HRT (7.5 d) from SRT (12 d). Probes of temperature, pH (METTLER TOLEDO, Switzerland), and ORP (PT4805-DPA-SC-S8/225, METTLER TOLEDO, Switzerland) were equipped for online monitoring.

All liquid samples were pre-filtered through 0.45 μm membrane (Whatman, the UK). Based on the chemical properties of soybean, a portion of amino acid and purine compound standards were selected for targeted analysis of the corresponding components in the pretreated samples. Samples were collected from day 104, 168, 200, 222, named Ⅰ, Ⅱ, Ⅲ and Ⅳ in following analysis of DON molecular characteristics

### Text S2 Analytical methods for basic physicochemical parameters

Dissolved organic carbon (DOC) was measured using a total organic carbon analyzer (TOC-L CPN, Shimadzu, Japan). $\text{NH}_{\text{4}}^{\text{+}}\text{-N}$ and TKN were tested by a Kjeldahl nitrogen analyzer (UDK-159, Velp Scientific Srl, Italy), respectively. Chemical oxygen demand (COD) was determined according to HACH method^[1]^. The concentration of lactic acid was quantified by ultra-high performance liquid chromatography (Premier UPLC; Waters, Milford, MA, USA) equipped with a UV detector and a Zorbax Stablebond Aq column (5 μm, $\text{4.6×150}$mm, Agilent, USA). The mobile phase for lactate analysis comprised 99.5% 20 mM NaH_2_PO_4_ and 0.5% acetonitrile, the flow rate was 1.0 mL/min, and the column temperature was 35°C. Short-chain carboxylates (including acetate, propionate, isobutyrate, *n*-butyrate, isovalerate, *n*-valerate), *n*-caproate, and alcohols were determined on a gas chromatograph (TRACE 1300, Thermo Fisher, USA) equipped with a flame ionization detector and a 30 m × 0.25 mm DB-WAX UI polyethylene glycol capillary column. Proportions of carbon dioxide, methane and hydrogen in collected gas were measured using a gas chromatography (Trace 1300/1310, Thermo Fisher Scientific, Waltham, MA, USA) equipped with a flame ionization detector and a thermal conductivity detector. A 1.8 m length × 2.1 mm I.D. Porapak N column (Supelco, St. Louis, USA) and a 0.5 m length × 2.1 mm S.S. HayeSep Q column (Supelco, St. Louis, USA) were used to filter and separate gas compositions.

### Text S3 Chemicals preparation

Anhydrous NaOH and high-performance liquid chromatography (HPLC) gradient grade acetonitrile, methanol, acetic acid solution, formic acid, acetate ammonium, dimethyl sulfoxide (DMSO), and ethyl acetate were bought from Sigma-Aldrich. HPLC grade 25% ammonia solution and amino acid standards (raceCERT®) was pruchased from Supelco. Lactate, xanthine, hypoxanthine, adenine, adenosine, guanosine, inosine, uracil, cytosine, xanthosine, thymine, thymidine, cytidine, uridine, adenosine monophosphate, cytidine monophosphate, uridine monophosphate, guanosine monophosphate, guanosine diphosphate, 2-Deoxyadenosine, and 2-Doxyguanosine were bought from Macklin.

### Text S4 Solutions preparation

The mixed AAS standards (2.5 mM, except 1.25 mM for Cysteine) were diluted to 0.05 - 50 μM (0.025 - 25 μM for Cysteine) using 70% (v/v) methanol. Tryptophan was prepared to concentrations of 0.2 - 10 mg/L with 70% (v/v) methanol. Stock solutions of xanthine, hypoxanthine, adenine, adenosine, guanosine, inosine, uracil, cytosine, and xanthosine were prepared in 0.1 M NaOH at a concentration of 1 mg/mL. The working solutions were prepared by diluting each individual stocks with 100% acetonitrile. Thymine, thymidine, cytidine, uridine, adenosine monophosphate, cytidine monophosphate, uridine monophosphate, guanosine monophosphate, guanosine diphosphate, 2-Deoxyadenosine, and 2-Doxyguanosine were prepared in milli-Q water and DMSO respectively. Additional nucleic acid-related compounds, including thymine, thymidine, cytidine, uridine, adenosine monophosphate (AMP), cytidine monophosphate (CMP), uridine monophosphate (UMP), guanosine monophosphate (GMP), guanosine diphosphate (GDP), 2-deoxyadenosine, and 2-deoxyguanosine, were also evaluated using Milli-Q water and DMSO as solvents. However, these compounds were not included in the final targeted analysis because they failed to meet the criteria for reliable quantification under the present analytical conditions. Specifically, these analytes exhibited inconsistent signal responses across the tested concentration range (S/N = 84-27,232 across 0.05-2 ppm), poor chromatographic reproducibility, and non-concentration-dependent responses, preventing robust calibration and reliable quantification. Therefore, they were excluded from subsequent quantitative analysis. Two acidic solutions (pH=2) were prepared by accurately adding 1.8 mL HCl (electronic pure grade) and 42.6 mL formic acid, respectively, into 2 L volumetric flasks and set the volume into 2 L with milli-Q water.

### Text S5 Samples preparation for targeted analysis

#### AAs analysis

Samples for the detection of AAs were directly diluted ten times with milli-Q water and methanol to obtain a final content of 70% methanol (v/v), and mixed by vortex for 60s to ensure the homogeneity.

#### Purine-derivatives analysis

Aliquoted 800 μL of acetonitrile and mixed with 200 μL of sample in a centrifuge tube before vertex for 60 s and centrifuge with a speed of 10000 rpm at 4 ℃ for 10 min for protein precipitation. Then, 500 μL of the supernatant was lyophilized and the residue was redissolved in 1000 μL with mixed solution (Text S6, mobile phase C and D at a volume ration of 96/4) for testing. The quality control sample of purine and its derived products was prepared by pooling 10 μL supernatant from each sample for monitoring the stability and reproducibility during sample analysis.

### Text S6 Liquid chromatography mass spectrometry

#### Instrument conditions of Liquid chromatography mass spectrometry

A triple quadrupole mass spectrometer and Qtrap system with an electrospray ionization (ESI) source coupled to a HPLC system (SCIEX Triple Quad 6500+, USA) was used for the separation and detection of AAs and prined-derivatives. Analytes were injected to the chromatography and separated on a BEH Amide column (2.1× 150 mm, 2.5 μm, Waters, USA). The minimum and maximum column pressure were set at 0.3 MPa and 60.0 MPa, respectively. Quantification was performed using multiple reaction monitoring (MRM) and the aera of specific substance was calculated using Analyst software 1.7.2. The column temperature was 40 °C and the sample chamber temperature was 4 °C during all analytical processes.

##### AAs analysis

Mobile phase A was milli-Q water with 2 mM ammonium acetate and 0.04% formic acid, and mobile phase B was 95% acetonitrile with 2 mM ammonium acetate and 0.04% formic acid. The flow rate was 0.4 ml/min and the injection volume was 1 µL. The gradient program took 15 min, including 4 min for equilibration and 11 min for separation and analysis. The gradient program was: 5%A, 0 to 1.2 min; ramp 5% to 20% A, 1.2 to 6 min; ramp 20% to 46% A, 6 to 6.1 min; ramp to 50% A, 6.1 to 9.5 min; incline to 5% A at 11.0 min and hold until 15 min. The ESI-MS spectra were acquired in positive ion multiple reaction monitoring (MRM) mode with a capillary voltage of 5.5 kV and a source temperature of 500 ℃. The retention time (RT) and MS information for each analyte including molecular weight (MW), MRM transitions, cone voltage (CV) and collision energies (CE) were shown in Table S3.

##### Purine analysis

Mobile phase C: 95% $\text{ACN-}\text{H}_{\text{2}}\text{O}$ (95:5, v/v) with 0.4% acetic acid and 10 mM ammonium acetate. Mobile phase D: 50% acetonitrile-$\text{H}_{\text{2}}\text{O}$ (1:1, v/v) with 0.1% formic acid and 20 mM ammonium acetate. The flow rate was 0.3 ml/min and the injection volume was 2 μL. The gradient program was: 6% D, 0 to 5 min; ramp 6% to 40% D, 5 to 7 min; ramp 40% to 57% D, 7 to 9 min; ramp to 95% D, 9 to 9.5 min; hold on for 1 min and ramp 95% to 40% D at 11 min; incline to 6% D at 11.5 min and hold until 15 min. The mass weight of precursor and product ion is shown in Table S3.

The mass spectrometer was operated in positive mode with an ESI spray voltage of 5.5 kV and a source temperature of 550℃. Ion source gas 1 and 2 were set at 50 psi. The curtain gas was set at 30.0 psi, and the collision gas was set at 8 psi. The RT and MS information for each analyte including molecular weight (MW), MRM transitions, CV and CE of nine purine-derivatives with satisfying response values on the standard curves were shown in Table S3.

The limits of detection (LOD) and quantification (LOQ) for each analyte were calculated based on the calibration curve approach, in accordance with ICH Q2(R1) guidelines. Calibration curves of AAs were established using seven concentration levels ranging from 0.5 to 10 mM (Fig.S2). For each compound, a linear regression model was fitted to the peak area-concentration data. LOD and LOQ were subsequently calculated and shown inTable S4. The LOD and LOQ for hypoxanthine were 13.27 μg/L and 40.22 μg/L, respectively, and for xanthine were 18.43 μg/L and 55.84 μg/L, respectively.

### Text S7 Solid phase extraction

DOM was extracted in triplicate via SPE (Supelco, USA) using two types of cartridges: manually filled mixed-sorbent cartridges (Mixed-SPE) and Bond Elut PPL cartridges (100 mg, 3 mL, Agilent) (PPL-SPE). All of the glassware was combusted for 5 h at 500℃ before SPE experiment. Each sample for SPE was diluted ten times with milli-Q water.

The procedure of using Bond Elut PPL cartridges was carried out in accordance with Qiu et al.^[2]^. Diluted samples were acidified to pH 2 with electronic pure HCl before desalting 3 mL diluted water-based mixture with Bond Elut PPL column (100 mg, 3mL, Agilent) as follows: SPE cartridges were conditioned with 6 mL methanol (HPLC grade, ≥99.9%, Sigma-Aldrich); 9 mL HCl-acidified water (pH 2) was passed through the cartridges to re-equilibrate with the aqueous phase; aliquots of 3 mL prepared sample were loaded onto the resins; 9 mL HCl solution (pH = 2) was used to wash the salt; after the cartridges were completely dried by air, DOM was eluted with 3 mL MeOH.

The procedures using mixed cartridges were modified from Li et al.^[3]^, without calibration of pH 2 using formic acid. Each mixed-SPE cartridge was manually filled with the 110 mg of Strata-XAW (33 μm, Phenomenex, USA), Strata-X-CW (33 μm, Phenomenex, USA), Bond Elut ENV (125 μm, Agilent, USA), and Oasis HLB (60 μm, Waters, USA) at a ratio of 1:1:1.5:2 in empty cartridges (3 mL, ANPEL, China).

Mixed-cartridges were conditioned with 4 mL methanol/ethylacetate (v/v 50:50) containing 2% ammonia followed by 2 mL methanol/ethylacetate (v/v 50:50) containing 1.7% formic acid. 6 mL formic acid solution (pH = 2) was passed through the cartridges, before 5 mL diluted sample were loaded onto the pre-conditioned and re-equilibrated resins at a flow rate of 6 mL/min. The pH 2 solution adjusted by formic acid was used to wash the salt. After air drying, DOM was sequentially eluted with 1 mL of methanol/ethylacetate (v/v 50:50) containing 2% ammonia followed by 0.5 mL of methanol/ethylacetate (v/v 50:50) containing 1.7% formic acid. Before analysis, aliquot certain amount of eluted mixture and dry it with pure nitrogen gas in a pre-cauterized glass tube at 30℃, and redissolved in MeOH to an equal volume subsequently. All prepared SPE eluates were transferred in amber glass vials and stored at -20 ℃ until analysis on UPLC Orbitrap MS/MS.

### Text S8 Operating conditions of Ultra-high Performance Liquid chromatography mass spectrometry

The molecular information was acquired with an ultra-performance liquid chromatography (Ultimate 3000, Thermo, USA) equipped with a Hypersil Gold C18 column (3 μm, 100 mm×2.1 mm) coupled to an Orbitrap mass analyzer (Q-Exactive plus, Thermo, USA) equipped with an ESI source. Chromatographic separation was performed with constant flow mode, and the flow rate was 0.3 mL/min. The sampler temperature was 4 ℃. Liquid chromatography mobile phase consisted of water (H_2_O) and acetonitrile (MeCN), both containing 10 mM ammonium acetate. The mobile phase started with H_2_O/MeCN = 80:20 (v/v) for the first 2 min, was then increased linearly to 20:80 in 5.5 min, and rose to 100% MeCN in 6.5 min, further lasting for 6 min. The gradient returned to the initial conditions within 2 min, and remained until the end. The total run time was 26 minutes. ESI parameters were as follows: sheath gas flow, ESI (+) and ESI (-): 45 L/min; auxiliary gas flow, ESI (+): 15 L/min, ESI (-): 10 L/min; capillary temperature, ESI (+) and ESI (-): 375 ℃; spray voltage, ESI (+): 3.5 kV, ESI (-): 3.2 kV. High-resolution mass spectrometry data were acquired in full-scan mode within the mass-to-charge (m/z) range of 100 - 1000 with a resolution of 140,000. MS/MS spectra with a resolution of 17500 were recorded in data-dependent acquisition mode, and the maximum ion injection time was set to 50 ms with automatic gain control targets of 10^6^. MS/MS precursor selection windows were set to 2.0 m/z.

### Text S9 Processing of mass data

The selection and identification of each compound were accomplished by the software Compound Discoverer 3.2 (Thermo Fisher Scientific, Inc., USA). Precursor ions were extracted from raw data with minimal intensity of 10^6^. The molecular mass weight of compounds ranged from 100 to 1000 Da and the mass tolerance was 5 ppm. The mass peaks of different samples, if RT drifting <0.05 min and mass discrepancy < 5 ppm, were merged. The mass peaks were abandoned, if occurring only in one of triplicate in the parallel test or appearing in the blank. The assignment range of molecular formula ranged from${}^{\text{12}}{\text{C}_{\text{2}}}{}^{\text{1}}{\text{H}_{\text{2}}}$ to ${}_{\text{ }}^{\text{12}}{\text{C}_{\text{50}}}{}^{\text{1}}{\text{H}_{\text{100}}}{}^{\text{1}}{\text{H}_{\text{100}}}{}^{\text{18}}{\text{O}_{\text{30}}}{}^{\text{14}}{\text{N}_{\text{2}}}{}^{\text{32}}{\text{S}_{\text{1}}}$, $\text{-10≤DBE-O≤10}$, $\text{0.33≤H/C≤2.5}$ and $\text{O/C≤1.2}$, and the tolerance for RT of peak was 0.05 min. Molecules were searched and further checked in databases including *ChemSpider* and *Pubchem* to guarantee the chemical possibility. The Venn diagram of the molecular formulas was produced by R package *Vennerable*. The subsequent step involved the classification of all identified DOM molecules according to their relative intensity. Molecules that presented in all samples and satisfy the intensity change between anaerobic and microaerophilic stages of 0.7 to 1.3 were defined as persistent components.

Structural confidence levels of assigned compounds were evaluated according to the framework of Schymanski et al.^[4]^ Given that MS/MS fragmentation data were not collected in this study, consistent with established HRMS-based approaches for population-level characterization of dissolved organic matter in complex matrices, the assignments correspond to Level 4 (unequivocal molecular formula assignment), supported by accurate mass measurement at ≤5 ppm tolerance, isotope pattern consistency, elemental composition constraints, and database cross-referencing against *ChemSpider* and *PubChem*. Structural-level assignments (Level 3 or above) were not claimed. All subsequent molecular-level analyses, including PMD, KMD, DBE, and AI_mod_ calculations, were performed at the molecular formula level, which is appropriate for and consistent with the confidence of assignment achieved^[5]^.

### Text S10 Calculations of molecular property indexes

The method of calculating double bond equivalence (DBE) referred to Chen et al. and specified in the following Eq.S1. The calculations of modified aromaticity index ($\text{AI}_{\text{mod}}$) referred to Koch and Dittmar^[6]^ and specified in the following Eq.S2. Nominal oxidation state of carbon (NOSC) was obtained from Zhang et al.^[7]^ and specified in the following Eq.S3. Symbols of elements below all referred to stoichiometric numbers of elements in each compound. Peak intensity weighted average DBE was calculated via Eq.S4. A_i_ represented the mass peak intensity of i-th DOM.

$$\text{DBE=1+C-}\frac{\text{1}}{\text{2}}\text{(H-N)}$$

Eq.S1

$$\text{AI}_{\text{mod}}\text{=}\frac{\text{1+C-}\frac{\text{O}}{\text{2}}\text{-S-}\frac{1}{\text{2}}(\text{N+H)}}{\text{C-}\frac{\text{O}}{\text{2}}\text{-N-S}}$$

Eq.S2

$$\text{NOSC=4-}\frac{\text{4C+H-2O-3N-2S}}{\text{C}}$$

Eq.S3

$$\text{DBE}_{\text{wa}}\text{ =}\sum_{\text{i}} \left[ \text{C}_{\text{i}}\text{+1-0.5}\left( \text{H}_{\text{i}}\text{-}\text{N}_{\text{i}} \right) \right]\text{⋅}\text{A}_{\text{i,j}}$$

Eq.S4

Gather *X*, *Y* and *Z* were the organic molecular formulas containing CH, CN and CO, respectively.

Note: $\text{AI}_{\text{mod}}$ was originally formulated for marine dissolved organic matter, where oxygen is assumed to occur predominantly in carboxyl groups. In the present fermentation system, contributions from amide and carbonyl oxygen may introduce uncertainty. $\text{AI}_{\text{mod}}$ values are therefore interpreted as relative indicators for comparative purposes only.

Kendrick mass (KM) and Kendrick mass defect (KMD) were calculated as follows:

$$\text{KM }\left( \text{NH}_{\text{2}} \right)\text{=}\frac{\text{m}}{\text{z}}\text{ ×}\frac{\text{nominal mass of }\text{NH}_{\text{2}}}{\text{exact mass of }\text{NH}_{\text{2}}}$$

Eq.S5

$$\text{KM }\left( \text{N}_{\text{2}} \right)\text{=}\frac{\text{m}}{\text{z}}\text{ ×}\frac{\text{nominal mass of }\text{N}_{\text{2}}}{\text{exact mass of }\text{N}_{\text{2}}}$$

Eq.S6

$$\text{KMD}\left( \text{NH}_{\text{2}} \right)\text{=nominal KM (}\text{NH}_{\text{2}}\text{)-KM (}\text{NH}_{\text{2}}\text{)}$$

Eq.S7

$$\text{KMD}\left( \text{N}_{\text{2}} \right)\text{=nominal KM (}\text{N}_{\text{2}}\text{)-KM (}\text{N}_{\text{2}}\text{)}$$

Eq.S8

Masses of assigned formula as well as corresponding RT are coupled into three groups (G1, G2, and G3) for PMD calculation. Directed paired mass distance (dPMD) method was adopted to couple direction inference and PMD analysis to extract the substrate–product relationships modified from Wang et al^[8]^. Chemical relationships were inferred by matching specific mass differences (PMDs) with known simple reactions from Table S5. Masses and formulas obtained were used for the validation of elemental composition and paired pathways. Inconsistent entries were excluded from further analysis. To further strengthen direction among all separate samples and build a flow of dPMD results, the recognized pathways were reconnected with overlap information of validated compounds.

### Text S11 DIA based proteomics

Protein digestion was performed in accordance with the standard procedure. Briefly, for each sample tube containing 100 μg protein, triethylammonium bicarbonate buffer (TEAB) and tris (2-carboxyethyl) phosphine were respectively added to the final concentration of 100 mM and 10 mM, then the tubes were cultivated at 37 ± 1°C for 1 h. Appropriate iodoacetamide was supplemented to the final concentration of 40 mM and the mixture was reacted at room temperature for 40 min in the dark. The surfactant was eliminated by centrifuging at 10000 g for 20 min, and precipitated protein was then resuspended with 100 µL of TEAB buffer (100 mM). According to the mass ratio of trypsin to protein of 1:50, the trypsin solution was added to each sample tube, and cultivated the tubes at 37 ± 1°C for 12 h. Bovine serum albumin was used as the standard, and the absorbance at 562 nm was read using a SPECTRA MAX microplate reader.

Peptides were separated and detected on an uPAC High Throughput column (75μm×5.5cm, Thermo, USA) using a VanquishNeo LC（Thermo, USA）system coupled to an Astral MS (Thermo, USA). The flow rate of mobile phase (Mobile phase G: 2% ACN-H_2_O (2:98, v/v) solution with 0.1% formic1802 acid; Mobile phase H: 80% ACN-H_2_O (80:20, v/v) solution with 0.1% formic acid) varied between 1.25 to 2.5 μL/min and achieved gradients of 4.8 min (180 samples per day, SPD). The gradient program took 8 min. All spectrum data were recorded in Thermo *Xcalibur* 4.7 (Thermo, USA).

The mass spectrometer was operated in positive mode with an ESI spray voltage of 1.5 kV. MS/MS spectra were recorded in data-independent (DIA) acquisition mode. Six peptides per protein and three daughter ions per peptide were chosen for quantification and the raw data were analyzed with Spectronaut™ 18/DIA-NN. Protein full discover rate (FDR) ≤ 0.01，peptide FDR≤ 0.01，peptide Confidence ≥ 99% and XIC width ≤ 75 ppm were set to exclude shared peptide segments and modified peptide segments, and then the quantification result could be obtained by adding peak aeras of each fragmented ion together. DIA data were analyzed and only qualification and quantification information of proteins and peptides were acquired from National Center for Biotechnology Information (NCBI) and used to analyze DON in the system. Several analytical considerations were applied during data interpretation. Modified peptides, including methionine-oxidized variants, were excluded from quantification to avoid retention time and ionization shifts associated with oxidation. Peptide persistence was defined based on consistent detection across all samples rather than absolute intensity thresholds, with no imputation applied for missing values. Multinomial logistic regression was subsequently applied to the relative amino acid composition of classified peptide groups to identify compositional features associated with persistence, independent of absolute quantitative values. To assess whether sequence-dependent detection bias could account for the observed Met and Trp enrichment, the amino acid frequency distribution of the full detected peptide pool (n = 4,079) was examined (Fig. S13). Met and Trp ranked 19^th^ and 20^th^ among all 20 standard amino acids globally (1.4% and 1.2%, respectively), confirming that their enrichment is specific to the stable-ratio fraction rather than a reflection of global detection preference.

### Text S12 DIA based peptides analysis and model selection

Three peptides were selected for FMO and Fukui function analysis. The nonapeptide AILNMFGMK was designed by integrating the amino acid composition of the soybean substrate, the multinomial logistic regression coefficient matrix derived from the detected peptide dataset, and the nitrogen contribution values of individual amino acids, to construct a peptide model with representative amino acid composition for standardized cross-peptide comparison of Fukui indices. The two supplementary model peptides DWSFILK and DELDIVIPTIR were selected directly from the LC-MS/MS-identified peptide dataset based on the multinomial logistic regression coefficient matrix to provide contrasting statistical association patterns. DWSFILK was selected as the p2-positive model, as Trp carries the highest positive regression coefficient in p2 (e^+0.341^) and the largest negative coefficient in absolute value across the entire matrix in p1 (e^−1.075^), indicating category-specific positive association with p2 degradation and strong inhibitory relationship with p1. DELDIVIPTIR was selected as a statistically neutral model peptide, as all constituent residues carry absolute regression coefficient values below 0.1 across all three categories, indicating no statistically dominant association pattern. Both supplementary model peptides were verified tryptic fragments identified by LC-MS/MS and traceable to annotated soybean protein entries. FMO and Fukui calculations were performed to explore qualitative differences in local molecular reactivity descriptors across peptide contexts. HOMO-LUMO gaps of DWSFILK and DELDIVIPTIR were calculated as 5.14 and 5.93 eV, respectively. In both peptides, elevated Fukui index values were primarily localized to side-chain or terminal positions rather than peptide backbone amide bonds. These calculations are interpreted as a hypothesis-generating analysis intended to provide qualitative insight into potential differences in peptide reactivity, rather than a mechanistic explanation of peptide persistence across the detected peptide pool.

All structural optimizations and vibrational frequency analyses were conducted using Gaussian 16^[9]^ with the B3LYP-D3BJ functional^[10]^. The 6-31G* basis set^[11, 12]^ was employed for both geometry optimizations and vibrational frequency calculations. Subsequently, single-point energies and Fukui functions were calculated at the M062x-D3/def2-TZVP level of theory^[13]^. To ensure accuracy, these single-point energy and Fukui function calculations incorporated the SMD implicit solvent model, which comprehensively accounts for both polar and non-polar solvation effects. Frontier molecular orbital trajectories and Fukui function grid data were generated using Multiwfn version 3.8 (development version)^[14, 15]^, and exported in the “.cub” file format. Visualization of these data was performed using the VESTA software^[16]^.

### Supplementary of Figure List

Fig.S1 Schematic diagram of the fermentation reactor. The reactor was equipped with a mechanical stirrer, a water-jacket heating system for temperature control, an air inlet valve for switching between anaerobic and micro-aeration conditions, and an online monitoring probe of pH, DO, and temperature.

Fig.S2 Standard curves used for the calculation of concentrations of AAs. Intensity (peak area) is plotted against concentration (μM) for each analyte. Linear regression equations and R² values are shown in each panel. All R² values exceeded 0.94, with the majority above 0.99.

Fig.S3 Isosurface representations of the electronic properties for the modeled nonapeptide. a. Original structure. b. Highest Occupied Molecular Orbital c. Lowest Unoccupied Molecular Orbital d. Fukui *f ^-^* function map. e. Fukui *f^0^* function map. f. Fukui *f^+^* function map.

Fig.S4 Isosurface representations of Fukui functions for supplementary peptide models, calculated at the M062x-D3/def2-TZVP level with SMD implicit solvation (isovalue = 0.001). a. Optimized molecular geometry. b. Electrophilic Fukui function (*f^+^*), indicating sites susceptible to nucleophilic attack. c. Nucleophilic Fukui function (*f ^-^*), indicating sites susceptible to electrophilic attack; high *f ^-^* values are concentrated at the terminal Arg guanidinium nitrogen atoms (N92, *f ^-^* = 0.2255; N94, *f ^-^* = 0.1669). (d) Radical Fukui function (*f^0^*), indicating sites susceptible to radical attack. e. Dual descriptor (DD); cyan regions denote nucleophilic character (*f ^-^* dominant) and yellow-green regions denote electrophilic character (*f^+^* dominant). High reactivity indices are localized at terminal positions, with backbone amide bonds exhibiting uniformly low Fukui values, consistent with electronic resistance to nonspecific peptide bond cleavage. f. Optimized molecular geometry. g. Electrophilic Fukui function (*f^+^*); reactive sites are concentrated on the Trp indole ring carbons (C18, *f^+^* = 0.1177; C21, *f^+^* = 0.1107). h. Nucleophilic Fukui function (*f ^-^*); high values are similarly localized on the indole ring system (C14, *f ^-^* = 0.1188; C15, *f ^-^* = 0.1033). i. Radical Fukui function (*f^0^*); the indole ring carbons again dominate (C15, *f^0^* = 0.0996; C18, *f^0^* = 0.0985). j. Dual descriptor (DD).

Fig.S5 Concentrations of COD and DOC in the DON transformation reactor over the 224-day operation period. Circles represent measured values while shaded areas indicate the range across parallel replicates.

Fig.S6 Proportion of H_2_ and CO_2_ in head space of DON transformation reactor from day 25 to day 125.

Fig.S7 Number of molecular formulas assigned across four samples (Ⅰ, Ⅱ, Ⅲ and Ⅳ) under positive and negative ESI modes, using PPL-SPE (top) and Mix-SPE (bottom) solid-phase extraction. Orange and green segments indicate DON-containing and other formulas, respectively.

Fig.S8. Relative ratio assignment of molecular formulas containing CHONS, CHON, CHOS, and CHO elemental compositions, acquired under negative ESI mode for samples extracted by PPL-SPE and Mix-SPE solid-phase extraction methods across four sampling time points spanning anaerobic and microaerobic phases.

Fig.S9 Molecular characteristics of persistent DON compounds extracted by PPL-SPE and Mix-SPE. a. O/C ratio versus double bond equivalence (DBE). b. N/C ratio versus modified aromaticity index (AI_mod_). Bubble color indicates molecular weight range (blue: <100 Da; red: >800 Da) and bubble size indicates signal intensity (×10^9^).

Fig.S10 Relative abundance (%) of persistent DON molecular formulas grouped by elemental class under ESI (+) mode, for PPL-SPE (top) and Mix-SPE (bottom) extracts. Each panel shows the oxygen number distribution within the indicated elemental composition range. X-axis labels denote molecular richness.

Fig.S11 Relative abundance (%) of persistent DON molecular formulas grouped by elemental class under ESI (-) mode, for PPL-SPE (top) and Mix-SPE (bottom) extracts. Each panel shows the oxygen number distribution within the indicated elemental composition range. X-axis labels denote molecular richness.

Fig.S12 Molecular characteristics and potential transformation pathways of DON at the level of molecular formula obtained under ESI (-) acquisition mode using Mix-SPE pretreatment. a. Van Krevelen Diagram of molecular formulas. b. The distribution of transformation paired pathways. d1: Paired two anaerobic samples; d2: Paired anaerobic and microaerobic samples; d3: Paired two microaerobic samples.

Fig.S13 Amino acid composition of the detected peptide pool. Frequency (%) of each of the 20 standard amino acids across all peptides identified by LC-MS/DIA in this study (n = 4,079 peptides). Amino acids are ordered by decreasing frequency. Methionine (Met, M) and tryptophan (Trp, W), whose compositional enrichment in the stable-ratio peptide fraction was identified by multinomial logistic regression, ranked 19^th^ and 20^th^ out of 20 amino acids in the global detected pool, with frequencies of 1.4% and 1.2%, respectively.

Fig.S14 Concentrations of free amino acid (mg-C/L) in the reactor at 22 sampling time points over a 222-day monitoring period. Each panel shows one of 16 measured amino acids.

### Supplementary of Table List

Table S1 Operation parameters for ORP^*^ control

| Stage | Ⅰ | Ⅱ | Ⅲ |
| --- | --- | --- | --- |
| State | Anaerobic | Microaerobic | |
| Time (d) | 0-168 | 169-219 | 219-224 |
| Aeration (L/h) | 0 | 0.16 | 0.36 |
| ORP^*^ (mV) | <-400 mV | -220mV~-190mV | -170 mV~-150mV |

^*^ The ORP was real scale of online monitoring.

Table S2 The ion information of purine-derivatives

| No | Analyte | Structure | Formula | CAS* | Precursor ion (m/z) | Production ion (m/z) | RT (min) | CE*(eV) | Dwell time (ms) |
| --- | --- | --- | --- | --- | --- | --- | --- | --- | --- |
| 1 | Adenine |  | C_5_H_5_N_5_ | 73-24-5 | 136.0 | 119.1 | 2.898 | 30 | 15 |
| 2 | Guanine |  | C₅H₅N₅O | 73-40-5 | 152.0 | 135.1 | 7.327 | 27 | 15 |
| 3 | Xanthine |  | C_5_H_4_N_4_O_2_ | 69-89-6 | 153.0 | 136.2 | 4.403 | 23 | 15 |
| 4 | Hypoxanthine |  | C_5_H_4_N_4_O | 68-94-0 | 136.9 | 110.2 | 3.401 | 28 | 15 |
| 5 | Adenosine |  | C_10_H_13_N_5_O_4_ | 58-61-7 | 268.1 | 136.2 | 3.391 | 40 | 15 |
| 6 | Guanosine |  | C_10_H_13_N_5_O_5_ | 118-00-3 | 284.2 | 152.2 | 8.483 | 15 | 15 |
| 7 | Xanthosine |  | C_10_H_12_N_4_O_6_ | 146-80-5 | 285.1 | 153.1 | 9.803 | 15 | 15 |
| 8 | uracil |  | C_4_H_4_N_2_O_2_ | 66-22-8 | 113.0 | 70.0 | 1.85 | 15 | 15 |
| 9 | cytosine |  | C_4_H_5_N_3_O | 71-30-7 | 112.0 | 95.0 | 4.485 | 30 | 15 |

*CAS and CE are abbreviations of Chemical Abstract Service and Collision Energy, respectively.

Table S3 The ion information of AAs analysis

| Name | Mass weight | CAS No. | Abbreviation | | Formula | Precursor ion | Product ion | CE | Structure (2D) |
| --- | --- | --- | --- | --- | --- | --- | --- | --- | --- |
| Phenylalanine | 165.0790 | 63-91-2 | Phe | F | C_9_H_11_NO_2_ | 166.1 | 120.1 | 15 |  |
| Tryptophan | 204.0899 | 73-22-3 | Trp | W | C_3_H_7_NO_3_ | 205.1 | 146.0 | 20 |  |
| Leucine | 131.0946 | 61-90-5 | Leu | L | C_6_H_13_NO_2_ | 132.1 | 86.1 | 10 |  |
| Isoleucine | 131.0946 | 73-32-5 | Ile | I | C_6_H_13_NO_2_ | 132.1 | 86.1 | 10 |  |
| Methionine | 149.0511 | 63-68-3 | Met | M | C_5_H_11_NO_2_S | 150.1 | 104.0 | 10 |  |
| Valine | 117.0790 | 72-18-4 | Val | V | C_5_H_11_NO_2_ | 118.1 | 72.1 | 10 |  |
| Proline | 115.0633 | 147-85-3 | Pro | P | C_5_H_9_NO_2_ | 116.1 | 70.0 | 10 |  |
| Tyrosine | 181.0739 | 60-18-4 | Tyr | Y | C_9_H_11_NO_3_ | 182.1 | 136.0 | 20 |  |
| Cysteine | 121.0198 | 52-90-4 | Cys | C | C_3_H_7_NO_2_S | 122.0 | 59 | 20 |  |
| Alanine | 89.0477 | 56-41-7 | Ala | A | C_3_H_7_NO_2_ | 90.1 | 44.0 | 10 |  |
| Threonine | 119.0582 | 72-19-5 | Thr | T | C_4_H_9_NO_3_ | 120.1 | 74.0 | 20 |  |
| Glycine | 75.0320 | 56-40-6 | Gly | G | C_2_H_5_NO_2_ | 76.0 | 30.0 | 10 |  |
| Glutamic acid | 147.0532 | 56-86-0 | Glu | E | C_5_H_9_NO_4_ | 148.1 | 84.0 | 15 |  |
| Serine | 105.0426 | 56-45-1 | Ser | S | C_3_H_7_NO_3_ | 106.1 | 60.0 | 10 |  |
| Arginine | 174.1117 | 74-79-3 | Arg | R | C_6_H_14_N_4_O_2_ | 175.1 | 70.1 | 20 |  |
| Aspartic acid | 133.0375 | 56-84-8 | Asp | D | C_4_H_7_NO_4_ | 134.1 | 88.0 | 10 |  |
| Lysine | 146.1055 | 56-87-1 | Lys | K | C_6_H_14_N_2_O_2_ | 147.1 | 84.0 | 15 |  |
| Histidine | 155.0695 | 71-00-1 | His | H | C_6_H_9_N_3_O_2_ | 156.1 | 110.0 | 20 |  |

Table S4 Limits of detection (LOD) and quantification (LOQ) of AAs by LC-MS

| Component | R^2^ | LOD (μM) | LOQ (μM) |
| --- | --- | --- | --- |
| Alanine | 1.00 | 0.60 | 1.82 |
| Arginine | 0.99 | 1.38 | 4.18 |
| Aspartic acid | 0.94 | 3.16 | 9.59 |
| Cysteine | 0.99 | 1.10 | 3.33 |
| Glycine | 0.99 | 1.50 | 4.54 |
| Histidine | 0.98 | 1.71 | 5.17 |
| Isoleucine | 1.00 | 0.78 | 2.35 |
| Leucine | 1.00 | 0.86 | 2.60 |
| Lysine | 0.99 | 1.53 | 4.64 |
| Methionine | 1.00 | 0.74 | 2.24 |
| Phenylalanine | 1.00 | 0.71 | 2.17 |
| Serine | 0.98 | 1.87 | 5.66 |
| Threonine | 0.99 | 1.42 | 4.31 |
| Tryptophan | 1.00 | 0.49 | 1.49 |
| Tyrosine | 1.00 | 0.52 | 1.57 |
| Valine | 1.00 | 0.81 | 2.45 |

Table S5 Library for PMD calculation and confirmation

| PMD | Element or group change |
| --- | --- |
| 0.9840 | -OH+NH_2_ |
| 2.0156 | ± 2H |
| 13.9793 | O-2H |
| 14.0156 | ± CH_2_ |
| 17.0265 | ± NH_3_ |
| 18.0106 | ± H_2_O |
| 18.9946 | - CN + COOH |
| 24.9952 | - CN + H |
| 27.0109 | ± HCN |
| 27.9949 | ± CO |
| 28.0313 | ± C_2_H_4_ |
| 29.9980 | - NO |
| 31.9721 | ± S |
| 33.9877 | ± H_2_S |
| 43.9898 | ± CO_2_ |
| 15.9949 | ± O |
| 42.0106 | ± C_2_H_2_O |
| 79.9568 | ± SO_3_ |
| 15.0109 | ± NH |

Table S6 The concentration of proteins in samples (Ⅰ, Ⅱ, Ⅲ, and Ⅳ)

| Name of Samples | Concentration (μg/μL) | Total (μg) |
| --- | --- | --- |
| Ⅰ | 2.972 | 1189 |
| Ⅱ | 4.243 | 1697 |
| Ⅲ | 5.551 | 2220 |
| Ⅳ | 4.506 | 1802 |

Table S7 Site-specific Fukui indices for the reference nonapeptide AILNMFGMK, calculated at the M062x-D3/def2-TZVP level with SMD implicit solvation

| Atom |  | q(N) | q(N+1) | q(N-1) | *f^-^* | *f^+^* | *f^0^* | CDD |
| --- | --- | --- | --- | --- | --- | --- | --- | --- |
| 1 | (N ) | -0.2085 | -0.2086 | -0.2084 | 0.0001 | 0.0001 | 0.0001 | 0 |
| 2 | (C ) | 0.0182 | 0.0181 | 0.0183 | 0.0001 | 0.0001 | 0.0001 | 0 |
| 3 | (C ) | 0.1768 | 0.1767 | 0.1768 | 0 | 0 | 0 | 0 |
| 4 | (O ) | -0.2857 | -0.2855 | -0.2859 | -0.0002 | -0.0002 | -0.0002 | 0 |
| 5 | (H ) | 0.028 | 0.028 | 0.028 | 0 | 0 | 0 | 0 |
| 6 | (C ) | -0.0535 | -0.0536 | -0.0534 | 0.0001 | 0.0001 | 0.0001 | -0.0001 |
| 7 | (C ) | -0.0532 | -0.0533 | -0.0529 | 0.0003 | 0.0002 | 0.0002 | -0.0001 |
| 8 | (C ) | -0.0008 | -0.0011 | -0.0005 | 0.0003 | 0.0004 | 0.0003 | 0.0001 |
| 9 | (N ) | -0.1112 | -0.1118 | -0.1105 | 0.0007 | 0.0006 | 0.0007 | -0.0002 |
| 10 | (C ) | 0.1383 | 0.1373 | 0.1396 | 0.0013 | 0.001 | 0.0011 | -0.0003 |
| 11 | (N ) | -0.3597 | -0.3616 | -0.357 | 0.0027 | 0.0018 | 0.0023 | -0.0008 |
| 12 | (N ) | -0.1281 | -0.13 | -0.1265 | 0.0017 | 0.0018 | 0.0017 | 0.0002 |
| 13 | (N ) | -0.0841 | -0.0842 | -0.084 | 0.0001 | 0.0001 | 0.0001 | 0 |
| 14 | (C ) | 0.0269 | 0.0269 | 0.0269 | 0 | 0 | 0 | 0 |
| 15 | (C ) | 0.1761 | 0.176 | 0.1761 | 0 | 0 | 0 | 0 |
| 16 | (O ) | -0.3332 | -0.3332 | -0.3332 | 0 | 0 | 0 | 0 |
| 17 | (H ) | 0.1028 | 0.1027 | 0.1029 | 0.0001 | 0.0001 | 0.0001 | 0 |
| 18 | (C ) | -0.0488 | -0.0488 | -0.0487 | 0.0001 | 0 | 0 | 0 |
| 19 | (C ) | -0.0508 | -0.0508 | -0.0509 | 0 | 0 | 0 | 0 |
| 20 | (C ) | 0.2343 | 0.2343 | 0.2343 | 0 | 0 | 0 | 0 |
| 21 | (O ) | -0.3162 | -0.3164 | -0.316 | 0.0002 | 0.0002 | 0.0002 | 0 |
| 22 | (O ) | -0.184 | -0.1838 | -0.1841 | -0.0002 | -0.0001 | -0.0001 | 0 |
| 23 | (N ) | -0.07 | -0.07 | -0.0699 | 0 | 0 | 0 | 0 |
| 24 | (C ) | 0.0078 | 0.0078 | 0.0079 | 0 | 0 | 0 | 0 |
| 25 | (C ) | 0.1739 | 0.1739 | 0.1739 | 0 | 0 | 0 | 0 |
| 26 | (O ) | -0.3522 | -0.3523 | -0.3522 | 0 | 0 | 0 | 0 |
| 27 | (H ) | 0.1126 | 0.1125 | 0.1126 | 0 | 0 | 0 | 0 |
| 28 | (N ) | -0.0724 | -0.0724 | -0.0724 | 0 | 0 | 0 | 0 |
| 29 | (C ) | 0.033 | 0.033 | 0.033 | 0 | 0 | 0 | 0 |
| 30 | (C ) | 0.1659 | 0.1659 | 0.1659 | 0 | 0 | 0 | 0 |
| 31 | (O ) | -0.3558 | -0.3558 | -0.3557 | 0.0001 | 0.0001 | 0.0001 | 0 |
| 32 | (H ) | 0.1177 | 0.1178 | 0.1177 | 0 | 0 | 0 | 0 |
| 33 | (C ) | -0.0331 | -0.0331 | -0.0331 | 0 | 0 | 0 | 0 |
| 34 | (C ) | 0.2313 | 0.2313 | 0.2313 | 0 | 0 | 0 | 0 |
| 35 | (O ) | -0.3175 | -0.3175 | -0.3176 | 0 | 0 | 0 | 0 |
| 36 | (O ) | -0.1826 | -0.1826 | -0.1825 | 0 | 0 | 0 | 0 |
| 37 | (N ) | -0.0848 | -0.0848 | -0.0849 | 0 | 0 | 0 | 0 |
| 38 | (C ) | 0.0263 | 0.0263 | 0.0263 | 0 | 0 | 0 | 0 |
| 39 | (C ) | 0.1779 | 0.1779 | 0.178 | 0.0001 | 0 | 0.0001 | 0 |
| 40 | (O ) | -0.2944 | -0.2944 | -0.2943 | 0.0001 | 0 | 0.0001 | 0 |
| 41 | (H ) | 0.0993 | 0.0993 | 0.0993 | 0 | 0 | 0 | 0 |
| 42 | (C ) | -0.0494 | -0.0494 | -0.0494 | 0 | 0 | 0 | 0 |
| 43 | (C ) | -0.0455 | -0.0455 | -0.0455 | 0 | 0 | 0 | 0 |
| 44 | (S ) | -0.0395 | -0.0396 | -0.0394 | 0.0001 | 0.0001 | 0.0001 | 0 |
| 45 | (C ) | -0.0871 | -0.087 | -0.0871 | 0 | 0 | 0 | 0 |
| 46 | (N ) | -0.0649 | -0.0649 | -0.0649 | 0 | 0 | 0 | 0 |
| 47 | (C ) | 0.024 | 0.024 | 0.0241 | 0.0001 | 0 | 0.0001 | -0.0001 |
| 48 | (C ) | 0.1705 | 0.1703 | 0.1709 | 0.0004 | 0.0001 | 0.0003 | -0.0003 |
| 49 | (O ) | -0.3547 | -0.355 | -0.354 | 0.0007 | 0.0003 | 0.0005 | -0.0004 |
| 50 | (H ) | 0.0997 | 0.0997 | 0.0998 | 0.0001 | 0 | 0 | 0 |
| 51 | (C ) | -0.0464 | -0.0464 | -0.0464 | 0 | 0 | 0 | 0 |
| 52 | (C ) | 0.0018 | 0.0019 | 0.0018 | 0 | 0 | 0 | 0 |
| 53 | (C ) | -0.0488 | -0.0488 | -0.0487 | 0.0001 | 0.0001 | 0.0001 | 0 |
| 54 | (C ) | -0.0448 | -0.0449 | -0.0446 | 0.0001 | 0.0001 | 0.0001 | 0 |
| 55 | (C ) | -0.04 | -0.0401 | -0.0399 | 0.0001 | 0.0001 | 0.0001 | 0 |
| 56 | (C ) | -0.0383 | -0.0383 | -0.0382 | 0 | 0 | 0 | 0 |
| 57 | (C ) | -0.0396 | -0.0395 | -0.0397 | -0.0001 | -0.0001 | -0.0001 | 0 |
| 58 | (N ) | -0.0723 | -0.0723 | -0.0721 | 0.0002 | 0 | 0.0001 | -0.0002 |
| 59 | (C ) | 0.0268 | 0.0265 | 0.0277 | 0.0009 | 0.0003 | 0.0006 | -0.0006 |
| 60 | (C ) | 0.1785 | 0.1771 | 0.1824 | 0.004 | 0.0014 | 0.0027 | -0.0026 |
| 61 | (O ) | -0.3029 | -0.3048 | -0.2975 | 0.0054 | 0.0019 | 0.0036 | -0.0036 |
| 62 | (H ) | 0.0944 | 0.0943 | 0.0947 | 0.0003 | 0.0001 | 0.0002 | -0.0002 |
| 63 | (C ) | -0.0106 | -0.0107 | -0.0103 | 0.0003 | 0.0001 | 0.0002 | -0.0002 |
| 64 | (C ) | -0.0887 | -0.0889 | -0.0884 | 0.0003 | 0.0002 | 0.0003 | -0.0001 |
| 65 | (C ) | -0.0886 | -0.0888 | -0.0882 | 0.0004 | 0.0002 | 0.0003 | -0.0002 |
| 66 | (N ) | -0.0717 | -0.073 | -0.0671 | 0.0046 | 0.0012 | 0.0029 | -0.0034 |
| 67 | (C ) | 0.0246 | 0.0219 | 0.0358 | 0.0112 | 0.0027 | 0.0069 | -0.0085 |
| 68 | (C ) | 0.1718 | 0.1699 | 0.1721 | 0.0003 | 0.0019 | 0.0011 | 0.0017 |
| 69 | (O ) | -0.3659 | -0.3732 | -0.3566 | 0.0093 | 0.0073 | 0.0083 | -0.0019 |
| 70 | (H ) | 0.1354 | 0.1339 | 0.1397 | 0.0043 | 0.0015 | 0.0029 | -0.0028 |
| 71 | (C ) | -0.05 | -0.058 | -0.0285 | 0.0215 | 0.0081 | 0.0148 | -0.0134 |
| 72 | (C ) | -0.0517 | -0.0835 | 0.0794 | 0.1311 | 0.0318 | 0.0815 | -0.0992 |
| 73 | (C ) | 0.0026 | -0.0819 | 0.1181 | 0.1154 | 0.0845 | 0.1 | -0.0309 |
| 74 | (N ) | -0.0531 | -0.0799 | 0.0295 | 0.0827 | 0.0268 | 0.0547 | -0.0559 |
| 75 | (C ) | 0.0225 | -0.0111 | 0.0564 | 0.0339 | 0.0336 | 0.0338 | -0.0003 |
| 76 | (C ) | -0.0547 | -0.18 | 0.0065 | 0.0612 | 0.1253 | 0.0933 | 0.0642 |
| 77 | (C ) | -0.0553 | -0.1424 | 0.0173 | 0.0727 | 0.087 | 0.0799 | 0.0144 |
| 78 | (C ) | -0.0673 | -0.1261 | -0.0258 | 0.0415 | 0.0588 | 0.0501 | 0.0173 |
| 79 | (C ) | -0.0578 | -0.17 | 0.0169 | 0.0747 | 0.1123 | 0.0935 | 0.0376 |
| 80 | (C ) | -0.046 | -0.094 | -0.022 | 0.024 | 0.048 | 0.036 | 0.0241 |
| 81 | (N ) | -0.0716 | -0.0738 | -0.0694 | 0.0022 | 0.0022 | 0.0022 | 0 |
| 82 | (C ) | 0.0305 | 0.0266 | 0.0321 | 0.0016 | 0.0039 | 0.0028 | 0.0023 |
| 83 | (C ) | 0.2265 | 0.2136 | 0.2282 | 0.0017 | 0.0129 | 0.0073 | 0.0112 |
| 84 | (O ) | -0.1462 | -0.1549 | -0.1426 | 0.0036 | 0.0087 | 0.0062 | 0.0051 |
| 85 | (H ) | 0.0885 | 0.0868 | 0.0898 | 0.0014 | 0.0017 | 0.0015 | 0.0003 |
| 86 | (C ) | -0.0497 | -0.0516 | -0.0488 | 0.0008 | 0.0019 | 0.0014 | 0.0011 |
| 87 | (C ) | -0.0123 | -0.0127 | -0.0119 | 0.0003 | 0.0004 | 0.0004 | 0.0001 |
| 88 | (C ) | -0.0873 | -0.0878 | -0.087 | 0.0003 | 0.0005 | 0.0004 | 0.0002 |
| 89 | (C ) | -0.0945 | -0.095 | -0.0942 | 0.0003 | 0.0005 | 0.0004 | 0.0002 |
| 90 | (H ) | 0.0139 | 0.0139 | 0.0139 | 0 | 0 | 0 | -0.0001 |
| 91 | (H ) | 0.0361 | 0.036 | 0.0362 | 0.0001 | 0.0001 | 0.0001 | -0.0001 |
| 92 | (H ) | 0.028 | 0.0277 | 0.0283 | 0.0004 | 0.0002 | 0.0003 | -0.0001 |
| 93 | (H ) | 0.0258 | 0.0257 | 0.0261 | 0.0002 | 0.0001 | 0.0002 | -0.0001 |
| 94 | (H ) | 0.0279 | 0.0267 | 0.0283 | 0.0004 | 0.0012 | 0.0008 | 0.0008 |
| 95 | (H ) | 0.0348 | 0.0345 | 0.0352 | 0.0004 | 0.0003 | 0.0004 | 0 |
| 96 | (H ) | 0.1248 | 0.1241 | 0.1257 | 0.0009 | 0.0007 | 0.0008 | -0.0002 |
| 97 | (H ) | 0.1258 | 0.1178 | 0.1301 | 0.0042 | 0.008 | 0.0061 | 0.0038 |
| 98 | (H ) | 0.1156 | 0.1116 | 0.1189 | 0.0032 | 0.004 | 0.0036 | 0.0007 |
| 99 | (H ) | 0.0742 | 0.073 | 0.0751 | 0.0009 | 0.0011 | 0.001 | 0.0002 |
| 100 | (H ) | 0.04 | 0.0399 | 0.0402 | 0.0001 | 0.0001 | 0.0001 | -0.0001 |
| 101 | (H ) | 0.1048 | 0.1047 | 0.1049 | 0.0001 | 0.0001 | 0.0001 | 0 |
| 102 | (H ) | 0.1124 | 0.1123 | 0.1125 | 0.0001 | 0 | 0.0001 | 0 |
| 103 | (H ) | 0.0275 | 0.0274 | 0.0276 | 0.0001 | 0.0001 | 0.0001 | 0 |
| 104 | (H ) | 0.0427 | 0.0427 | 0.0428 | 0 | 0 | 0 | 0 |
| 105 | (H ) | 0.0477 | 0.0478 | 0.0476 | -0.0001 | -0.0001 | -0.0001 | 0 |
| 106 | (H ) | 0.0442 | 0.0443 | 0.0441 | -0.0001 | -0.0001 | -0.0001 | 0 |
| 107 | (H ) | 0.0325 | 0.0325 | 0.0325 | 0 | 0 | 0 | 0 |
| 108 | (H ) | 0.0588 | 0.0588 | 0.0588 | 0 | 0 | 0 | 0 |
| 109 | (H ) | 0.068 | 0.0679 | 0.068 | 0 | 0 | 0 | 0 |
| 110 | (H ) | 0.072 | 0.072 | 0.072 | 0 | 0 | 0 | 0 |
| 111 | (H ) | 0.0654 | 0.0655 | 0.0654 | 0 | 0 | 0 | 0 |
| 112 | (H ) | 0.0524 | 0.0523 | 0.0524 | 0 | 0 | 0 | 0 |
| 113 | (H ) | 0.039 | 0.039 | 0.039 | 0 | 0 | 0 | 0 |
| 114 | (H ) | 0.0438 | 0.0438 | 0.0438 | 0 | 0 | 0 | 0 |
| 115 | (H ) | 0.0514 | 0.0514 | 0.0514 | 0 | 0 | 0 | 0 |
| 116 | (H ) | 0.0331 | 0.033 | 0.0332 | 0.0001 | 0.0001 | 0.0001 | 0 |
| 117 | (H ) | 0.0481 | 0.0481 | 0.0482 | 0 | 0 | 0 | 0 |
| 118 | (H ) | 0.047 | 0.047 | 0.0469 | 0 | 0 | 0 | 0 |
| 119 | (H ) | 0.0395 | 0.0395 | 0.0395 | 0.0001 | 0 | 0 | 0 |
| 120 | (H ) | 0.0504 | 0.0505 | 0.0503 | -0.0001 | -0.0001 | -0.0001 | 0 |
| 121 | (H ) | 0.0413 | 0.0412 | 0.0414 | 0.0001 | 0 | 0.0001 | 0 |
| 122 | (H ) | 0.0444 | 0.0444 | 0.0445 | 0.0001 | 0 | 0.0001 | 0 |
| 123 | (H ) | 0.0526 | 0.0527 | 0.0524 | -0.0002 | -0.0001 | -0.0001 | 0 |
| 124 | (H ) | 0.0418 | 0.0417 | 0.0419 | 0.0001 | 0.0001 | 0.0001 | 0 |
| 125 | (H ) | 0.0379 | 0.0379 | 0.038 | 0 | 0 | 0 | 0 |
| 126 | (H ) | 0.056 | 0.056 | 0.0561 | 0.0001 | 0.0001 | 0.0001 | 0 |
| 127 | (H ) | 0.0243 | 0.0243 | 0.0244 | 0 | 0 | 0 | 0 |
| 128 | (H ) | 0.0323 | 0.0321 | 0.0327 | 0.0004 | 0.0002 | 0.0003 | -0.0002 |
| 129 | (H ) | 0.0395 | 0.0392 | 0.0402 | 0.0006 | 0.0003 | 0.0005 | -0.0003 |
| 130 | (H ) | 0.0213 | 0.021 | 0.0216 | 0.0003 | 0.0003 | 0.0003 | 0 |
| 131 | (H ) | 0.0341 | 0.0339 | 0.0346 | 0.0005 | 0.0002 | 0.0003 | -0.0003 |
| 132 | (H ) | 0.0391 | 0.0389 | 0.0395 | 0.0004 | 0.0002 | 0.0003 | -0.0002 |
| 133 | (H ) | 0.0309 | 0.0308 | 0.0311 | 0.0002 | 0.0001 | 0.0002 | -0.0002 |
| 134 | (H ) | 0.023 | 0.0229 | 0.0233 | 0.0003 | 0.0001 | 0.0002 | -0.0001 |
| 135 | (H ) | 0.047 | 0.0467 | 0.048 | 0.0009 | 0.0003 | 0.0006 | -0.0006 |
| 136 | (H ) | 0.0392 | 0.0301 | 0.0621 | 0.0229 | 0.0091 | 0.016 | -0.0138 |
| 137 | (H ) | 0.046 | 0.0364 | 0.0673 | 0.0213 | 0.0096 | 0.0154 | -0.0116 |
| 138 | (H ) | 0.0738 | 0.0335 | 0.1184 | 0.0446 | 0.0403 | 0.0425 | -0.0043 |
| 139 | (H ) | 0.1689 | 0.1498 | 0.2073 | 0.0385 | 0.0191 | 0.0288 | -0.0194 |
| 140 | (H ) | 0.0388 | -0.0121 | 0.0674 | 0.0287 | 0.0509 | 0.0398 | 0.0223 |
| 141 | (H ) | 0.0419 | 0.0103 | 0.0647 | 0.0229 | 0.0316 | 0.0272 | 0.0087 |
| 142 | (H ) | 0.0582 | 0.0018 | 0.0857 | 0.0275 | 0.0564 | 0.042 | 0.0289 |
| 143 | (H ) | 0.0492 | 0.0064 | 0.0796 | 0.0304 | 0.0429 | 0.0366 | 0.0125 |
| 144 | (H ) | 0.0316 | 0.0274 | 0.0395 | 0.0079 | 0.0042 | 0.006 | -0.0037 |
| 145 | (H ) | 0.0164 | 0.0152 | 0.0171 | 0.0007 | 0.0012 | 0.001 | 0.0005 |
| 146 | (H ) | 0.0395 | 0.0377 | 0.0405 | 0.0009 | 0.0019 | 0.0014 | 0.0009 |
| 147 | (H ) | 0.0276 | 0.0268 | 0.028 | 0.0004 | 0.0007 | 0.0006 | 0.0003 |
| 148 | (H ) | 0.0325 | 0.032 | 0.0328 | 0.0003 | 0.0005 | 0.0004 | 0.0001 |
| 149 | (H ) | 0.0325 | 0.0322 | 0.0327 | 0.0002 | 0.0004 | 0.0003 | 0.0001 |
| 150 | (H ) | 0.0308 | 0.0304 | 0.031 | 0.0002 | 0.0004 | 0.0003 | 0.0002 |
| 151 | (H ) | 0.0333 | 0.0328 | 0.0337 | 0.0004 | 0.0006 | 0.0005 | 0.0002 |
| 152 | (H ) | 0.0239 | 0.0234 | 0.0242 | 0.0003 | 0.0005 | 0.0004 | 0.0002 |
| 153 | (H ) | 0.0181 | 0.0176 | 0.0183 | 0.0002 | 0.0005 | 0.0003 | 0.0003 |
| 154 | (H ) | 0.0466 | 0.0424 | 0.0488 | 0.0022 | 0.0042 | 0.0032 | 0.002 |
| 155 | (O ) | -0.3108 | -0.3283 | -0.305 | 0.0058 | 0.0175 | 0.0117 | 0.0118 |
| 156 | (H ) | 0.219 | 0.219 | 0.219 | 0 | 0 | 0 | 0 |
| 157 | (H ) | 0.2049 | 0.2049 | 0.2049 | 0 | 0 | 0 | 0 |
| 158 | (H ) | 0.1777 | 0.1627 | 0.1845 | 0.0069 | 0.0149 | 0.0109 | 0.0081 |

Table S8 Site-specific Fukui indices for the supplementary peptide model DELDIVIPTIR, calculated at the M062x-D3/def2-TZVP level with SMD implicit solvation

| Atom | q(N) | q(N+1) | q(N-1) | *f^-^* | *f^+^* | *f^0^* | CDD |
| --- | --- | --- | --- | --- | --- | --- | --- |
| 1(N) | -0.2131 | -0.2289 | -0.2125 | 0.0006 | 0.0159 | 0.0082 | 0.0153 |
| 2(C) | 0.0234 | -0.0017 | 0.0241 | 0.0007 | 0.0250 | 0.0129 | 0.0243 |
| 3(C) | 0.1850 | 0.0488 | 0.1854 | 0.0004 | 0.1363 | 0.0683 | 0.1359 |
| 4(O) | -0.2387 | -0.3310 | -0.2381 | 0.0005 | 0.0923 | 0.0464 | 0.0917 |
| 5(C) | -0.0535 | -0.0760 | -0.0517 | 0.0018 | 0.0225 | 0.0121 | 0.0207 |
| 6(C) | 0.2122 | 0.1727 | 0.2161 | 0.0040 | 0.0395 | 0.0217 | 0.0355 |
| 7(O) | -0.3291 | -0.3680 | -0.3225 | 0.0066 | 0.0389 | 0.0228 | 0.0323 |
| 8(N) | -0.0490 | -0.1093 | -0.0486 | 0.0004 | 0.0603 | 0.0304 | 0.0599 |
| 9(C) | 0.0401 | 0.0185 | 0.0402 | 0.0001 | 0.0215 | 0.0108 | 0.0214 |
| 10(C) | 0.1595 | 0.1146 | 0.1596 | 0.0001 | 0.0450 | 0.0225 | 0.0449 |
| 11(O) | -0.3686 | -0.4182 | -0.3683 | 0.0003 | 0.0496 | 0.0249 | 0.0493 |
| 12(H) | 0.1513 | 0.1228 | 0.1515 | 0.0002 | 0.0285 | 0.0144 | 0.0282 |
| 13(C) | -0.0476 | -0.0559 | -0.0475 | 0.0001 | 0.0083 | 0.0042 | 0.0082 |
| 14(C) | -0.0423 | -0.0484 | -0.0423 | 0.0000 | 0.0061 | 0.0031 | 0.0061 |
| 15(C) | 0.2243 | 0.2112 | 0.2243 | 0.0000 | 0.0131 | 0.0066 | 0.0131 |
| 16(O) | -0.3322 | -0.3452 | -0.3320 | 0.0002 | 0.0130 | 0.0066 | 0.0129 |
| 17(N) | -0.0768 | -0.0968 | -0.0768 | 0.0000 | 0.0199 | 0.0100 | 0.0199 |
| 18(C) | 0.0268 | 0.0213 | 0.0268 | 0.0000 | 0.0054 | 0.0027 | 0.0054 |
| 19(C) | 0.1593 | 0.1543 | 0.1593 | 0.0000 | 0.0049 | 0.0025 | 0.0049 |
| 20(O) | -0.3628 | -0.3690 | -0.3628 | 0.0000 | 0.0063 | 0.0031 | 0.0063 |
| 21(H) | 0.1057 | 0.0905 | 0.1057 | 0.0000 | 0.0152 | 0.0076 | 0.0152 |
| 22(C) | -0.0459 | -0.0487 | -0.0459 | 0.0000 | 0.0027 | 0.0014 | 0.0027 |
| 23(C) | -0.0126 | -0.0143 | -0.0126 | 0.0000 | 0.0017 | 0.0009 | 0.0017 |
| 24(C) | -0.0932 | -0.0945 | -0.0931 | 0.0001 | 0.0013 | 0.0007 | 0.0012 |
| 25(C) | -0.0902 | -0.0912 | -0.0901 | 0.0000 | 0.0010 | 0.0005 | 0.0010 |
| 26(N) | -0.0641 | -0.0672 | -0.0641 | 0.0000 | 0.0031 | 0.0016 | 0.0030 |
| 27(C) | 0.0282 | 0.0272 | 0.0282 | 0.0000 | 0.0010 | 0.0005 | 0.0009 |
| 28(C) | 0.1576 | 0.1572 | 0.1577 | 0.0000 | 0.0005 | 0.0002 | 0.0004 |
| 29(O) | -0.3725 | -0.3736 | -0.3724 | 0.0000 | 0.0012 | 0.0006 | 0.0011 |
| 30(H) | 0.1595 | 0.1573 | 0.1595 | 0.0000 | 0.0021 | 0.0011 | 0.0021 |
| 31(C) | -0.0370 | -0.0376 | -0.0370 | 0.0000 | 0.0005 | 0.0003 | 0.0005 |
| 32(C) | 0.2268 | 0.2265 | 0.2268 | 0.0000 | 0.0003 | 0.0001 | 0.0002 |
| 33(O) | -0.3248 | -0.3250 | -0.3248 | 0.0000 | 0.0002 | 0.0001 | 0.0002 |
| 34(N) | -0.0671 | -0.0674 | -0.0671 | 0.0000 | 0.0003 | 0.0001 | 0.0003 |
| 35(C) | 0.0296 | 0.0295 | 0.0296 | 0.0000 | 0.0001 | 0.0001 | 0.0001 |
| 36(C) | 0.1732 | 0.1730 | 0.1732 | 0.0000 | 0.0001 | 0.0001 | 0.0001 |
| 37(O) | -0.3366 | -0.3369 | -0.3365 | 0.0000 | 0.0003 | 0.0002 | 0.0003 |
| 38(H) | 0.1288 | 0.1284 | 0.1288 | 0.0000 | 0.0004 | 0.0002 | 0.0004 |
| 39(C) | -0.0148 | -0.0149 | -0.0148 | 0.0000 | 0.0001 | 0.0001 | 0.0001 |
| 40(C) | -0.0527 | -0.0528 | -0.0527 | 0.0000 | 0.0001 | 0.0000 | 0.0000 |
| 41(C) | -0.0880 | -0.0881 | -0.0880 | 0.0000 | 0.0001 | 0.0000 | 0.0001 |
| 42(C) | -0.0900 | -0.0901 | -0.0900 | 0.0000 | 0.0001 | 0.0000 | 0.0001 |
| 43(N) | -0.0539 | -0.0539 | -0.0539 | 0.0000 | 0.0000 | 0.0000 | 0.0000 |
| 44(C) | 0.0269 | 0.0268 | 0.0269 | 0.0000 | 0.0001 | 0.0001 | 0.0001 |
| 45(C) | 0.1619 | 0.1618 | 0.1619 | 0.0000 | 0.0001 | 0.0001 | 0.0001 |
| 46(O) | -0.3803 | -0.3808 | -0.3801 | 0.0002 | 0.0005 | 0.0003 | 0.0003 |
| 47(H) | 0.1538 | 0.1536 | 0.1539 | 0.0000 | 0.0002 | 0.0001 | 0.0002 |
| 48(C) | -0.0095 | -0.0099 | -0.0095 | 0.0000 | 0.0003 | 0.0002 | 0.0003 |
| 49(C) | -0.0968 | -0.0971 | -0.0968 | 0.0000 | 0.0003 | 0.0002 | 0.0003 |
| 50(C) | -0.0963 | -0.0990 | -0.0962 | 0.0001 | 0.0028 | 0.0014 | 0.0027 |
| 51(N) | -0.0627 | -0.0628 | -0.0627 | 0.0000 | 0.0001 | 0.0000 | 0.0001 |
| 52(C) | 0.0336 | 0.0336 | 0.0337 | 0.0000 | 0.0001 | 0.0000 | 0.0000 |
| 53(C) | 0.1753 | 0.1752 | 0.1753 | 0.0000 | 0.0001 | 0.0001 | 0.0000 |
| 54(O) | -0.2922 | -0.2921 | -0.2925 | -0.0003 | -0.0001 | -0.0002 | 0.0002 |
| 55(H) | 0.0992 | 0.0991 | 0.0992 | 0.0001 | 0.0000 | 0.0000 | 0.0000 |
| 56(C) | 0.0243 | 0.0243 | 0.0243 | 0.0000 | 0.0000 | 0.0000 | 0.0000 |
| 57(C) | -0.0533 | -0.0534 | -0.0533 | 0.0000 | 0.0000 | 0.0000 | 0.0000 |
| 58(C) | -0.0881 | -0.0881 | -0.0880 | 0.0000 | 0.0000 | 0.0000 | 0.0000 |
| 59(C) | -0.0871 | -0.0872 | -0.0871 | 0.0000 | 0.0001 | 0.0001 | 0.0000 |
| 60(N) | -0.0147 | -0.0148 | -0.0147 | 0.0001 | 0.0001 | 0.0001 | 0.0000 |
| 61(C) | 0.0261 | 0.0261 | 0.0261 | 0.0000 | 0.0000 | 0.0000 | 0.0001 |
| 62(C) | 0.1554 | 0.1553 | 0.1554 | 0.0000 | 0.0000 | 0.0000 | 0.0001 |
| 63(O) | -0.3837 | -0.3837 | -0.3840 | -0.0004 | 0.0000 | -0.0002 | 0.0004 |
| 64(C) | -0.0400 | -0.0401 | -0.0400 | 0.0000 | 0.0000 | 0.0000 | 0.0000 |
| 65(C) | -0.0392 | -0.0392 | -0.0391 | 0.0000 | 0.0000 | 0.0000 | 0.0000 |
| 66(C) | 0.0160 | 0.0159 | 0.0161 | 0.0001 | 0.0001 | 0.0001 | 0.0000 |
| 67(N) | -0.0714 | -0.0714 | -0.0713 | 0.0001 | 0.0001 | 0.0001 | -0.0001 |
| 68(C) | 0.0243 | 0.0242 | 0.0244 | 0.0001 | 0.0001 | 0.0001 | 0.0001 |
| 69(C) | 0.1633 | 0.1628 | 0.1634 | 0.0002 | 0.0005 | 0.0003 | 0.0003 |
| 70(O) | -0.3635 | -0.3640 | -0.3631 | 0.0004 | 0.0005 | 0.0005 | 0.0002 |
| 71(H) | 0.1353 | 0.1352 | 0.1355 | 0.0002 | 0.0001 | 0.0001 | -0.0001 |
| 72(C) | 0.0623 | 0.0622 | 0.0623 | 0.0001 | 0.0001 | 0.0001 | 0.0000 |
| 73(O) | -0.2649 | -0.2650 | -0.2648 | 0.0000 | 0.0001 | 0.0001 | 0.0000 |
| 74(C) | -0.0960 | -0.0961 | -0.0960 | 0.0000 | 0.0000 | 0.0000 | 0.0000 |
| 75(N) | -0.0751 | -0.0752 | -0.0749 | 0.0001 | 0.0001 | 0.0001 | 0.0000 |
| 76(C) | 0.0347 | 0.0343 | 0.0351 | 0.0004 | 0.0004 | 0.0004 | -0.0001 |
| 77(C) | 0.1771 | 0.1752 | 0.1784 | 0.0013 | 0.0019 | 0.0016 | 0.0005 |
| 78(O) | -0.2707 | -0.2721 | -0.2688 | 0.0019 | 0.0014 | 0.0016 | -0.0005 |
| 79(H) | 0.0852 | 0.0851 | 0.0853 | 0.0001 | 0.0001 | 0.0001 | 0.0000 |
| 80(C) | -0.0104 | -0.0105 | -0.0102 | 0.0002 | 0.0001 | 0.0001 | -0.0001 |
| 81(C) | -0.0599 | -0.0599 | -0.0598 | 0.0001 | 0.0000 | 0.0001 | -0.0001 |
| 82(C) | -0.0556 | -0.0557 | -0.0556 | 0.0000 | 0.0001 | 0.0001 | 0.0000 |
| 83(C) | -0.0912 | -0.0913 | -0.0910 | 0.0002 | 0.0002 | 0.0002 | 0.0000 |
| 84(N) | -0.0711 | -0.0720 | -0.0698 | 0.0013 | 0.0009 | 0.0011 | -0.0003 |
| 85(C) | 0.0302 | 0.0281 | 0.0342 | 0.0040 | 0.0021 | 0.0030 | -0.0019 |
| 86(C) | 0.2091 | 0.2002 | 0.2102 | 0.0011 | 0.0089 | 0.0050 | 0.0078 |
| 87(O) | -0.3145 | -0.3250 | -0.3112 | 0.0033 | 0.0105 | 0.0069 | 0.0073 |
| 88(H) | 0.1506 | 0.1496 | 0.1522 | 0.0015 | 0.0010 | 0.0013 | -0.0005 |
| 89(C) | -0.0391 | -0.0406 | -0.0297 | 0.0094 | 0.0014 | 0.0054 | -0.0080 |
| 90(C) | -0.0576 | -0.0586 | -0.0303 | 0.0273 | 0.0009 | 0.0141 | -0.0264 |
| 91(C) | -0.0187 | -0.0194 | 0.0254 | 0.0442 | 0.0007 | 0.0224 | -0.0435 |
| 92(N) | -0.2624 | -0.2636 | -0.0370 | 0.2255 | 0.0012 | 0.1133 | -0.2243 |
| 93(C) | 0.1362 | 0.1354 | 0.2346 | 0.0984 | 0.0008 | 0.0496 | -0.0976 |
| 94(N) | -0.1830 | -0.1837 | -0.0161 | 0.1669 | 0.0007 | 0.0838 | -0.1662 |
| 95(N) | -0.1038 | -0.1051 | -0.0470 | 0.0568 | 0.0012 | 0.0290 | -0.0556 |
| 96(H) | 0.0433 | 0.0227 | 0.0446 | 0.0013 | 0.0206 | 0.0110 | 0.0193 |
| 97(H) | 0.0527 | 0.0336 | 0.0541 | 0.0014 | 0.0191 | 0.0102 | 0.0177 |
| 98(H) | 0.0435 | 0.0118 | 0.0442 | 0.0006 | 0.0318 | 0.0162 | 0.0311 |
| 99(H) | 0.1199 | 0.1094 | 0.1202 | 0.0004 | 0.0104 | 0.0054 | 0.0101 |
| 100(H) | 0.1231 | 0.1119 | 0.1236 | 0.0005 | 0.0112 | 0.0059 | 0.0107 |
| 101(H) | 0.0399 | 0.0298 | 0.0400 | 0.0001 | 0.0101 | 0.0051 | 0.0099 |
| 102(H) | 0.0380 | 0.0285 | 0.0382 | 0.0001 | 0.0095 | 0.0048 | 0.0094 |
| 103(H) | 0.0569 | 0.0513 | 0.0568 | -0.0001 | 0.0056 | 0.0028 | 0.0057 |
| 104(H) | 0.0621 | 0.0540 | 0.0621 | -0.0001 | 0.0081 | 0.0040 | 0.0082 |
| 105(H) | 0.0572 | 0.0267 | 0.0573 | 0.0001 | 0.0305 | 0.0153 | 0.0304 |
| 106(H) | 0.0400 | 0.0363 | 0.0401 | 0.0001 | 0.0037 | 0.0019 | 0.0036 |
| 107(H) | 0.0294 | 0.0267 | 0.0294 | 0.0000 | 0.0027 | 0.0014 | 0.0028 |
| 108(H) | 0.0240 | 0.0194 | 0.0240 | 0.0000 | 0.0046 | 0.0023 | 0.0046 |
| 109(H) | 0.0268 | 0.0258 | 0.0268 | -0.0001 | 0.0011 | 0.0005 | 0.0011 |
| 110(H) | 0.0179 | 0.0172 | 0.0180 | 0.0001 | 0.0008 | 0.0004 | 0.0007 |
| 111(H) | 0.0287 | 0.0274 | 0.0289 | 0.0001 | 0.0013 | 0.0007 | 0.0012 |
| 112(H) | 0.0298 | 0.0288 | 0.0298 | 0.0000 | 0.0010 | 0.0005 | 0.0009 |
| 113(H) | 0.0311 | 0.0301 | 0.0311 | -0.0001 | 0.0010 | 0.0005 | 0.0011 |
| 114(H) | 0.0295 | 0.0280 | 0.0296 | 0.0001 | 0.0015 | 0.0008 | 0.0014 |
| 115(H) | 0.0482 | 0.0421 | 0.0483 | 0.0001 | 0.0061 | 0.0031 | 0.0061 |
| 116(H) | 0.0731 | 0.0725 | 0.0732 | 0.0000 | 0.0006 | 0.0003 | 0.0006 |
| 117(H) | 0.0645 | 0.0640 | 0.0645 | 0.0000 | 0.0005 | 0.0003 | 0.0005 |
| 118(H) | 0.0434 | 0.0423 | 0.0434 | 0.0000 | 0.0011 | 0.0005 | 0.0010 |
| 119(H) | 0.0265 | 0.0264 | 0.0265 | 0.0000 | 0.0001 | 0.0001 | 0.0001 |
| 120(H) | 0.0362 | 0.0361 | 0.0362 | 0.0000 | 0.0001 | 0.0001 | 0.0001 |
| 121(H) | 0.0194 | 0.0193 | 0.0194 | 0.0000 | 0.0000 | 0.0000 | 0.0000 |
| 122(H) | 0.0317 | 0.0317 | 0.0317 | 0.0000 | 0.0001 | 0.0000 | 0.0001 |
| 123(H) | 0.0218 | 0.0217 | 0.0218 | 0.0000 | 0.0001 | 0.0000 | 0.0000 |
| 124(H) | 0.0231 | 0.0231 | 0.0232 | 0.0000 | 0.0001 | 0.0000 | 0.0001 |
| 125(H) | 0.0308 | 0.0308 | 0.0309 | 0.0000 | 0.0001 | 0.0000 | 0.0000 |
| 126(H) | 0.0297 | 0.0296 | 0.0297 | 0.0000 | 0.0000 | 0.0000 | 0.0000 |
| 127(H) | 0.0302 | 0.0301 | 0.0302 | 0.0000 | 0.0001 | 0.0000 | 0.0001 |
| 128(H) | 0.0453 | 0.0451 | 0.0453 | 0.0000 | 0.0002 | 0.0001 | 0.0002 |
| 129(H) | 0.0354 | 0.0348 | 0.0355 | 0.0001 | 0.0006 | 0.0003 | 0.0004 |
| 130(H) | 0.0291 | 0.0270 | 0.0290 | 0.0000 | 0.0020 | 0.0010 | 0.0021 |
| 131(H) | 0.0165 | 0.0146 | 0.0167 | 0.0001 | 0.0019 | 0.0010 | 0.0018 |
| 132(H) | 0.0240 | 0.0210 | 0.0240 | 0.0000 | 0.0030 | 0.0015 | 0.0030 |
| 133(H) | 0.0318 | 0.0316 | 0.0317 | -0.0001 | 0.0002 | 0.0000 | 0.0003 |
| 134(H) | 0.0165 | 0.0162 | 0.0165 | 0.0000 | 0.0003 | 0.0001 | 0.0003 |
| 135(H) | 0.0265 | 0.0260 | 0.0266 | 0.0001 | 0.0005 | 0.0003 | 0.0005 |
| 136(H) | 0.0377 | 0.0377 | 0.0377 | 0.0000 | 0.0000 | 0.0000 | -0.0001 |
| 137(H) | 0.0512 | 0.0512 | 0.0512 | 0.0000 | 0.0000 | 0.0000 | 0.0001 |
| 138(H) | 0.0420 | 0.0419 | 0.0421 | 0.0001 | 0.0001 | 0.0001 | 0.0000 |
| 139(H) | 0.0428 | 0.0429 | 0.0428 | -0.0001 | 0.0000 | 0.0000 | 0.0000 |
| 140(H) | 0.0499 | 0.0498 | 0.0500 | 0.0001 | 0.0001 | 0.0001 | 0.0000 |
| 141(H) | 0.0466 | 0.0465 | 0.0468 | 0.0001 | 0.0001 | 0.0001 | 0.0000 |
| 142(H) | 0.0584 | 0.0584 | 0.0585 | 0.0001 | 0.0000 | 0.0000 | 0.0000 |
| 143(H) | 0.0465 | 0.0465 | 0.0463 | -0.0002 | 0.0000 | -0.0001 | 0.0002 |
| 144(H) | 0.0416 | 0.0415 | 0.0417 | 0.0001 | 0.0001 | 0.0001 | 0.0000 |
| 145(H) | 0.0417 | 0.0416 | 0.0418 | 0.0001 | 0.0001 | 0.0001 | 0.0000 |
| 146(H) | 0.0307 | 0.0307 | 0.0308 | 0.0000 | 0.0000 | 0.0000 | 0.0000 |
| 147(H) | 0.0329 | 0.0329 | 0.0329 | 0.0000 | 0.0000 | 0.0000 | 0.0001 |
| 148(H) | 0.1886 | 0.1885 | 0.1887 | 0.0001 | 0.0001 | 0.0001 | 0.0000 |
| 149(H) | 0.0474 | 0.0472 | 0.0474 | 0.0000 | 0.0002 | 0.0001 | 0.0002 |
| 150(H) | 0.0293 | 0.0276 | 0.0362 | 0.0069 | 0.0016 | 0.0043 | -0.0053 |
| 151(H) | 0.0488 | 0.0471 | 0.0565 | 0.0077 | 0.0017 | 0.0047 | -0.0060 |
| 152(H) | 0.0281 | 0.0270 | 0.0459 | 0.0178 | 0.0010 | 0.0094 | -0.0168 |
| 153(H) | 0.0180 | 0.0167 | 0.0342 | 0.0163 | 0.0012 | 0.0087 | -0.0150 |
| 154(H) | 0.0246 | 0.0238 | 0.0541 | 0.0295 | 0.0009 | 0.0152 | -0.0286 |
| 155(H) | 0.0283 | 0.0275 | 0.0798 | 0.0514 | 0.0008 | 0.0261 | -0.0506 |
| 156(H) | 0.1330 | 0.1325 | 0.1873 | 0.0544 | 0.0005 | 0.0274 | -0.0539 |
| 157(H) | 0.1426 | 0.1423 | 0.2007 | 0.0581 | 0.0003 | 0.0292 | -0.0577 |
| 158(H) | 0.1406 | 0.1393 | 0.1689 | 0.0283 | 0.0013 | 0.0148 | -0.0270 |
| 159(H) | 0.1537 | 0.1527 | 0.1873 | 0.0336 | 0.0011 | 0.0173 | -0.0325 |
| 160(H) | 0.0485 | 0.0453 | 0.0516 | 0.0031 | 0.0032 | 0.0031 | 0.0002 |
| 161(H) | 0.0482 | 0.0480 | 0.0482 | 0.0001 | 0.0001 | 0.0001 | 0.0001 |
| 162(H) | 0.0177 | 0.0178 | 0.0177 | 0.0000 | -0.0001 | 0.0000 | -0.0001 |
| 163(H) | 0.0247 | 0.0246 | 0.0247 | 0.0000 | 0.0001 | 0.0001 | 0.0000 |
| 164(H) | 0.0310 | 0.0310 | 0.0311 | 0.0000 | 0.0000 | 0.0000 | 0.0000 |
| 165(H) | 0.0308 | 0.0308 | 0.0308 | 0.0000 | 0.0000 | 0.0000 | 0.0000 |
| 166(H) | 0.0291 | 0.0291 | 0.0292 | 0.0000 | 0.0001 | 0.0001 | 0.0001 |
| 167(H) | 0.0356 | 0.0355 | 0.0357 | 0.0001 | 0.0001 | 0.0001 | 0.0000 |
| 168(H) | 0.0348 | 0.0347 | 0.0348 | 0.0000 | 0.0001 | 0.0000 | 0.0000 |
| 169(H) | 0.0362 | 0.0362 | 0.0363 | 0.0000 | 0.0001 | 0.0001 | 0.0000 |
| 170(H) | 0.0481 | 0.0478 | 0.0485 | 0.0003 | 0.0004 | 0.0003 | 0.0000 |
| 171(H) | 0.0371 | 0.0369 | 0.0372 | 0.0002 | 0.0001 | 0.0001 | 0.0000 |
| 172(H) | 0.0278 | 0.0276 | 0.0280 | 0.0002 | 0.0001 | 0.0002 | -0.0001 |
| 173(H) | 0.0097 | 0.0098 | 0.0097 | 0.0001 | -0.0001 | 0.0000 | -0.0001 |
| 174(H) | 0.0320 | 0.0318 | 0.0321 | 0.0001 | 0.0001 | 0.0001 | 0.0000 |
| 175(H) | 0.0199 | 0.0198 | 0.0199 | 0.0000 | 0.0001 | 0.0000 | 0.0000 |
| 176(H) | 0.0300 | 0.0299 | 0.0300 | 0.0001 | 0.0001 | 0.0001 | 0.0000 |
| 177(H) | 0.0285 | 0.0283 | 0.0286 | 0.0001 | 0.0002 | 0.0002 | 0.0001 |
| 178(H) | 0.0340 | 0.0339 | 0.0343 | 0.0002 | 0.0002 | 0.0002 | -0.0001 |
| 179(O) | -0.1749 | -0.1752 | -0.1749 | 0.0000 | 0.0003 | 0.0002 | 0.0003 |
| 180(H) | 0.2180 | 0.2178 | 0.2180 | 0.0000 | 0.0002 | 0.0001 | 0.0002 |
| 181(O) | -0.1835 | -0.1906 | -0.1836 | -0.0001 | 0.0071 | 0.0035 | 0.0072 |
| 182(H) | 0.2133 | 0.2099 | 0.2134 | 0.0000 | 0.0035 | 0.0018 | 0.0034 |
| 183(O) | -0.2079 | -0.2321 | -0.1965 | 0.0114 | 0.0242 | 0.0178 | 0.0128 |
| 184(H) | 0.1183 | 0.1111 | 0.1287 | 0.0104 | 0.0072 | 0.0088 | -0.0032 |
| 185(O) | -0.1840 | -0.1949 | -0.1822 | 0.0018 | 0.0109 | 0.0063 | 0.0092 |
| 186(H) | 0.1322 | 0.1195 | 0.1329 | 0.0007 | 0.0127 | 0.0067 | 0.0120 |

Table S9 Site-specific Fukui indices for the supplementary peptide model DWSFILK, calculated at the M062x-D3/def2-TZVP level with SMD implicit solvation

| **Atom** | **q(N)** | **q(N+1)** | **q(N-1)** | **f-** | **f+** | **f0** | **CDD** |
| --- | --- | --- | --- | --- | --- | --- | --- |
| 1(N) | -0.2153 | -0.2168 | -0.2142 | 0.0011 | 0.0015 | 0.0013 | 0.0004 |
| 2(C) | 0.0209 | 0.0200 | 0.0217 | 0.0007 | 0.0009 | 0.0008 | 0.0002 |
| 3(C) | 0.1761 | 0.1714 | 0.1786 | 0.0024 | 0.0047 | 0.0036 | 0.0023 |
| 4(O) | -0.2767 | -0.2811 | -0.2732 | 0.0035 | 0.0044 | 0.0040 | 0.0009 |
| 5(C) | -0.0453 | -0.0458 | -0.0451 | 0.0003 | 0.0005 | 0.0004 | 0.0003 |
| 6(C) | 0.2198 | 0.2192 | 0.2200 | 0.0003 | 0.0005 | 0.0004 | 0.0003 |
| 7(O) | -0.3334 | -0.3342 | -0.3328 | 0.0006 | 0.0008 | 0.0007 | 0.0002 |
| 8(N) | -0.0581 | -0.0600 | -0.0570 | 0.0012 | 0.0019 | 0.0015 | 0.0007 |
| 9(C) | 0.0316 | 0.0268 | 0.0425 | 0.0109 | 0.0047 | 0.0078 | -0.0061 |
| 10(C) | 0.1705 | 0.1630 | 0.1740 | 0.0035 | 0.0075 | 0.0055 | 0.0039 |
| 11(O) | -0.2774 | -0.2847 | -0.2718 | 0.0056 | 0.0073 | 0.0064 | 0.0016 |
| 12(H) | 0.1588 | 0.1561 | 0.1622 | 0.0033 | 0.0027 | 0.0030 | -0.0006 |
| 13(C) | -0.0361 | -0.0466 | -0.0164 | 0.0197 | 0.0105 | 0.0151 | -0.0092 |
| 14(C) | -0.0484 | -0.0895 | 0.0704 | 0.1188 | 0.0412 | 0.0800 | -0.0776 |
| 15(C) | -0.0095 | -0.1054 | 0.0938 | 0.1033 | 0.0959 | 0.0996 | -0.0074 |
| 16(N) | -0.0489 | -0.0805 | 0.0369 | 0.0858 | 0.0317 | 0.0587 | -0.0541 |
| 17(C) | 0.0225 | -0.0123 | 0.0545 | 0.0320 | 0.0348 | 0.0334 | 0.0028 |
| 18(C) | -0.0638 | -0.1815 | 0.0155 | 0.0793 | 0.1177 | 0.0985 | 0.0384 |
| 19(C) | -0.0618 | -0.1498 | 0.0141 | 0.0759 | 0.0880 | 0.0820 | 0.0121 |
| 20(C) | -0.0658 | -0.1262 | -0.0141 | 0.0516 | 0.0604 | 0.0560 | 0.0088 |
| 21(C) | -0.0537 | -0.1644 | 0.0303 | 0.0840 | 0.1107 | 0.0974 | 0.0267 |
| 22(C) | -0.0398 | -0.0870 | -0.0107 | 0.0291 | 0.0473 | 0.0382 | 0.0182 |
| 23(N) | -0.0711 | -0.0751 | -0.0677 | 0.0034 | 0.0041 | 0.0037 | 0.0006 |
| 24(C) | 0.0246 | 0.0234 | 0.0257 | 0.0010 | 0.0012 | 0.0011 | 0.0001 |
| 25(C) | 0.1668 | 0.1654 | 0.1670 | 0.0002 | 0.0014 | 0.0008 | 0.0012 |
| 26(O) | -0.2759 | -0.2781 | -0.2749 | 0.0010 | 0.0022 | 0.0016 | 0.0011 |
| 27(H) | 0.1327 | 0.1305 | 0.1343 | 0.0016 | 0.0022 | 0.0019 | 0.0005 |
| 28(C) | 0.0347 | 0.0340 | 0.0352 | 0.0005 | 0.0007 | 0.0006 | 0.0001 |
| 29(O) | -0.2377 | -0.2384 | -0.2373 | 0.0005 | 0.0006 | 0.0006 | 0.0002 |
| 30(N) | -0.0676 | -0.0681 | -0.0674 | 0.0002 | 0.0006 | 0.0004 | 0.0004 |
| 31(C) | 0.0302 | 0.0300 | 0.0304 | 0.0002 | 0.0003 | 0.0002 | 0.0001 |
| 32(C) | 0.1596 | 0.1592 | 0.1599 | 0.0003 | 0.0004 | 0.0004 | 0.0001 |
| 33(O) | -0.3641 | -0.3651 | -0.3633 | 0.0008 | 0.0010 | 0.0009 | 0.0002 |
| 34(H) | 0.1151 | 0.1147 | 0.1153 | 0.0002 | 0.0005 | 0.0003 | 0.0003 |
| 35(C) | -0.0413 | -0.0415 | -0.0412 | 0.0001 | 0.0002 | 0.0002 | 0.0001 |
| 36(C) | -0.0003 | -0.0003 | -0.0003 | 0.0000 | 0.0000 | 0.0000 | 0.0000 |
| 37(C) | -0.0414 | -0.0415 | -0.0414 | 0.0000 | 0.0000 | 0.0000 | 0.0000 |
| 38(C) | -0.0416 | -0.0417 | -0.0416 | 0.0001 | 0.0001 | 0.0001 | 0.0001 |
| 39(C) | -0.0471 | -0.0472 | -0.0469 | 0.0001 | 0.0002 | 0.0002 | 0.0000 |
| 40(C) | -0.0383 | -0.0384 | -0.0381 | 0.0002 | 0.0002 | 0.0002 | 0.0000 |
| 41(C) | -0.0411 | -0.0412 | -0.0409 | 0.0001 | 0.0001 | 0.0001 | 0.0000 |
| 42(N) | -0.0714 | -0.0714 | -0.0715 | 0.0000 | 0.0000 | 0.0000 | 0.0001 |
| 43(C) | 0.0253 | 0.0248 | 0.0256 | 0.0004 | 0.0004 | 0.0004 | 0.0001 |
| 44(C) | 0.1503 | 0.1501 | 0.1503 | 0.0001 | 0.0001 | 0.0001 | 0.0001 |
| 45(O) | -0.3827 | -0.3837 | -0.3819 | 0.0008 | 0.0010 | 0.0009 | 0.0002 |
| 46(H) | 0.1085 | 0.1082 | 0.1086 | 0.0001 | 0.0003 | 0.0002 | 0.0002 |
| 47(C) | -0.0105 | -0.0123 | -0.0095 | 0.0010 | 0.0018 | 0.0014 | 0.0008 |
| 48(C) | -0.0489 | -0.0497 | -0.0482 | 0.0007 | 0.0008 | 0.0008 | 0.0001 |
| 49(C) | -0.0928 | -0.0960 | -0.0898 | 0.0030 | 0.0031 | 0.0031 | 0.0001 |
| 50(C) | -0.0959 | -0.0993 | -0.0937 | 0.0022 | 0.0034 | 0.0028 | 0.0012 |
| 51(N) | -0.0723 | -0.0725 | -0.0724 | -0.0001 | 0.0001 | 0.0000 | 0.0002 |
| 52(C) | 0.0243 | 0.0242 | 0.0244 | 0.0001 | 0.0001 | 0.0001 | 0.0001 |
| 53(C) | 0.1483 | 0.1482 | 0.1484 | 0.0001 | 0.0002 | 0.0001 | 0.0001 |
| 54(O) | -0.3892 | -0.3897 | -0.3889 | 0.0003 | 0.0004 | 0.0004 | 0.0001 |
| 55(H) | 0.1045 | 0.1039 | 0.1047 | 0.0002 | 0.0006 | 0.0004 | 0.0005 |
| 56(C) | -0.0471 | -0.0476 | -0.0468 | 0.0003 | 0.0004 | 0.0004 | 0.0001 |
| 57(C) | -0.0135 | -0.0149 | -0.0127 | 0.0008 | 0.0014 | 0.0011 | 0.0005 |
| 58(C) | -0.0910 | -0.0924 | -0.0904 | 0.0006 | 0.0014 | 0.0010 | 0.0008 |
| 59(C) | -0.0881 | -0.0892 | -0.0873 | 0.0008 | 0.0011 | 0.0010 | 0.0003 |
| 60(N) | -0.0790 | -0.0792 | -0.0789 | 0.0001 | 0.0002 | 0.0002 | 0.0001 |
| 61(C) | 0.0273 | 0.0272 | 0.0273 | 0.0001 | 0.0001 | 0.0001 | 0.0000 |
| 62(C) | 0.2060 | 0.2059 | 0.2060 | 0.0001 | 0.0001 | 0.0001 | 0.0000 |
| 63(H) | 0.1034 | 0.1032 | 0.1035 | 0.0001 | 0.0002 | 0.0001 | 0.0001 |
| 64(C) | -0.0420 | -0.0421 | -0.0419 | 0.0001 | 0.0001 | 0.0001 | 0.0000 |
| 65(C) | -0.0503 | -0.0504 | -0.0503 | 0.0000 | 0.0001 | 0.0001 | 0.0000 |
| 66(C) | -0.0566 | -0.0566 | -0.0565 | 0.0001 | 0.0001 | 0.0001 | 0.0000 |
| 67(C) | -0.0155 | -0.0155 | -0.0154 | 0.0000 | 0.0000 | 0.0000 | 0.0000 |
| 68(N) | -0.2459 | -0.2460 | -0.2459 | 0.0000 | 0.0001 | 0.0000 | 0.0000 |
| 69(H) | 0.0691 | 0.0683 | 0.0693 | 0.0002 | 0.0007 | 0.0005 | 0.0005 |
| 70(H) | 0.0580 | 0.0576 | 0.0583 | 0.0003 | 0.0004 | 0.0003 | 0.0001 |
| 71(H) | 0.0398 | 0.0388 | 0.0408 | 0.0010 | 0.0010 | 0.0010 | 0.0000 |
| 72(H) | 0.1165 | 0.1159 | 0.1170 | 0.0005 | 0.0006 | 0.0005 | 0.0002 |
| 73(H) | 0.0946 | 0.0939 | 0.0951 | 0.0005 | 0.0007 | 0.0006 | 0.0002 |
| 74(H) | 0.0472 | 0.0363 | 0.0672 | 0.0200 | 0.0109 | 0.0154 | -0.0091 |
| 75(H) | 0.0535 | 0.0431 | 0.0715 | 0.0181 | 0.0103 | 0.0142 | -0.0077 |
| 76(H) | 0.0699 | 0.0301 | 0.1096 | 0.0397 | 0.0398 | 0.0398 | 0.0001 |
| 77(H) | 0.1736 | 0.1541 | 0.2089 | 0.0353 | 0.0195 | 0.0274 | -0.0158 |
| 78(H) | 0.0417 | 0.0002 | 0.0693 | 0.0275 | 0.0415 | 0.0345 | 0.0140 |
| 79(H) | 0.0439 | 0.0164 | 0.0673 | 0.0234 | 0.0275 | 0.0254 | 0.0041 |
| 80(H) | 0.0550 | 0.0065 | 0.0860 | 0.0310 | 0.0484 | 0.0397 | 0.0174 |
| 81(H) | 0.0476 | 0.0091 | 0.0779 | 0.0304 | 0.0385 | 0.0344 | 0.0081 |
| 82(H) | 0.0468 | 0.0430 | 0.0528 | 0.0060 | 0.0038 | 0.0049 | -0.0022 |
| 83(H) | 0.0558 | 0.0550 | 0.0562 | 0.0005 | 0.0007 | 0.0006 | 0.0003 |
| 84(H) | 0.0474 | 0.0467 | 0.0481 | 0.0007 | 0.0007 | 0.0007 | 0.0001 |
| 85(H) | 0.1491 | 0.1488 | 0.1493 | 0.0003 | 0.0003 | 0.0003 | 0.0001 |
| 86(H) | 0.0569 | 0.0556 | 0.0580 | 0.0011 | 0.0013 | 0.0012 | 0.0002 |
| 87(H) | 0.0507 | 0.0505 | 0.0509 | 0.0002 | 0.0003 | 0.0002 | 0.0001 |
| 88(H) | 0.0378 | 0.0376 | 0.0379 | 0.0001 | 0.0002 | 0.0002 | 0.0001 |
| 89(H) | 0.0504 | 0.0503 | 0.0505 | 0.0001 | 0.0001 | 0.0001 | 0.0000 |
| 90(H) | 0.0545 | 0.0544 | 0.0546 | 0.0001 | 0.0001 | 0.0001 | 0.0000 |
| 91(H) | 0.0527 | 0.0526 | 0.0527 | 0.0000 | 0.0001 | 0.0001 | 0.0000 |
| 92(H) | 0.0561 | 0.0560 | 0.0562 | 0.0001 | 0.0001 | 0.0001 | 0.0000 |
| 93(H) | 0.0534 | 0.0533 | 0.0535 | 0.0001 | 0.0001 | 0.0001 | 0.0000 |
| 94(H) | 0.0462 | 0.0457 | 0.0465 | 0.0003 | 0.0004 | 0.0004 | 0.0001 |
| 95(H) | 0.0271 | 0.0224 | 0.0289 | 0.0017 | 0.0047 | 0.0032 | 0.0030 |
| 96(H) | 0.0257 | 0.0201 | 0.0292 | 0.0036 | 0.0056 | 0.0046 | 0.0020 |
| 97(H) | 0.0238 | 0.0211 | 0.0256 | 0.0018 | 0.0027 | 0.0023 | 0.0009 |
| 98(H) | 0.0292 | 0.0275 | 0.0305 | 0.0014 | 0.0016 | 0.0015 | 0.0003 |
| 99(H) | 0.0175 | 0.0165 | 0.0183 | 0.0008 | 0.0010 | 0.0009 | 0.0001 |
| 100(H) | 0.0294 | 0.0282 | 0.0306 | 0.0012 | 0.0012 | 0.0012 | 0.0000 |
| 101(H) | 0.0295 | 0.0276 | 0.0311 | 0.0017 | 0.0018 | 0.0017 | 0.0002 |
| 102(H) | 0.0248 | 0.0201 | 0.0273 | 0.0024 | 0.0048 | 0.0036 | 0.0024 |
| 103(H) | 0.0204 | 0.0158 | 0.0250 | 0.0046 | 0.0046 | 0.0046 | 0.0000 |
| 104(H) | 0.0398 | 0.0390 | 0.0405 | 0.0007 | 0.0008 | 0.0008 | 0.0001 |
| 105(H) | 0.0385 | 0.0377 | 0.0390 | 0.0006 | 0.0007 | 0.0007 | 0.0002 |
| 106(H) | 0.0251 | 0.0246 | 0.0254 | 0.0003 | 0.0005 | 0.0004 | 0.0001 |
| 107(H) | 0.0192 | 0.0162 | 0.0204 | 0.0012 | 0.0030 | 0.0021 | 0.0018 |
| 108(H) | 0.0302 | 0.0275 | 0.0308 | 0.0006 | 0.0027 | 0.0017 | 0.0020 |
| 109(H) | 0.0239 | 0.0232 | 0.0243 | 0.0004 | 0.0007 | 0.0006 | 0.0003 |
| 110(H) | 0.0281 | 0.0270 | 0.0288 | 0.0007 | 0.0011 | 0.0009 | 0.0004 |
| 111(H) | 0.0316 | 0.0297 | 0.0328 | 0.0012 | 0.0019 | 0.0016 | 0.0006 |
| 112(H) | 0.0280 | 0.0270 | 0.0287 | 0.0008 | 0.0010 | 0.0009 | 0.0002 |
| 113(H) | 0.0327 | 0.0320 | 0.0332 | 0.0005 | 0.0007 | 0.0006 | 0.0002 |
| 114(H) | 0.0376 | 0.0374 | 0.0376 | 0.0001 | 0.0001 | 0.0001 | 0.0000 |
| 115(H) | 0.0395 | 0.0394 | 0.0396 | 0.0001 | 0.0001 | 0.0001 | 0.0000 |
| 116(H) | 0.0299 | 0.0299 | 0.0300 | 0.0001 | 0.0001 | 0.0001 | 0.0000 |
| 117(H) | 0.0170 | 0.0169 | 0.0170 | 0.0000 | 0.0000 | 0.0000 | 0.0000 |
| 118(H) | 0.0306 | 0.0305 | 0.0307 | 0.0001 | 0.0001 | 0.0001 | 0.0000 |
| 119(H) | 0.0279 | 0.0278 | 0.0279 | 0.0001 | 0.0001 | 0.0001 | 0.0000 |
| 120(H) | 0.0237 | 0.0236 | 0.0237 | 0.0000 | 0.0000 | 0.0000 | 0.0000 |
| 121(H) | 0.0240 | 0.0240 | 0.0240 | 0.0000 | 0.0000 | 0.0000 | 0.0000 |
| 122(H) | 0.0266 | 0.0266 | 0.0267 | 0.0000 | 0.0001 | 0.0001 | 0.0000 |
| 123(H) | 0.0904 | 0.0904 | 0.0904 | 0.0000 | 0.0000 | 0.0000 | 0.0000 |
| 124(H) | 0.0994 | 0.0993 | 0.0995 | 0.0001 | 0.0001 | 0.0001 | 0.0000 |
| 125(H) | 0.0471 | 0.0469 | 0.0471 | 0.0001 | 0.0001 | 0.0001 | 0.0000 |
| 126(O) | -0.3366 | -0.3366 | -0.3367 | 0.0000 | 0.0000 | 0.0000 | 0.0000 |
| 127(O) | -0.1734 | -0.1735 | -0.1733 | 0.0001 | 0.0001 | 0.0001 | 0.0000 |
| 128(H) | 0.2174 | 0.2173 | 0.2174 | 0.0000 | 0.0001 | 0.0000 | 0.0000 |
| 129(O) | -0.1771 | -0.1774 | -0.1769 | 0.0002 | 0.0003 | 0.0002 | 0.0002 |
| 130(H) | 0.2158 | 0.2155 | 0.2160 | 0.0002 | 0.0003 | 0.0003 | 0.0001 |

Table S10 Estimated nitrogen contributions of quantifiable dissolved organic nitrogen (DON) fractions at representative sampling time points across anaerobic and microaerobic phases

| Samples | Proteins* (g-N /L) | AAs (g-N/L) | Purine-derivatives (g-N/L) | Total concentration (g-N/L) | DON% |
| --- | --- | --- | --- | --- | --- |
| Ⅰ | 0.476 | 0.009 | 0.033 | 0.518 | **70.7%** |
| Ⅱ | 0.679 | 0.014 | 0.045 | 0.738 | **63.0%** |
| Ⅲ | 0.888 | 0.018 | 0.056 | 0.962 | **68.1%** |
| Ⅳ | 0.721 | 0.015 | 0.030 | 0.766 | **66.0%** |

* Protein-N was estimated by multiplying total dissolved protein concentration (determined by BCA assay) by a nitrogen conversion factor of 0.16 (i.e., assuming 16% nitrogen content by mass, equivalent to a Kjeldahl conversion factor of 6.25).

### Reference

(1)	Rice, E.W., L. Bridgewater and A.P.H. Association. *Standard methods for the examination of water and wastewater*. Vol. 10.(American public health association Washington, DC, 2012).

(2)	Qiu, J., F. Lü, H. Zhang, et al. UPLC Orbitrap MS/MS-based fingerprints of dissolved organic matter in waste leachate driven by waste age. *Journal of Hazardous Materials*, **383** (2020).

(3)	Li, T., F. Lü, H. Zhang, et al. Nontarget insights into the fate of Cl^-^/Br^-^ containing DOM in leachate during membrane treatment. *Environmental Science & Technology*, **57**(42): 16033-16042 (2023).

(4)	Schymanski, E.L., J. Jeon, R. Gulde, et al. Identifying small molecules via high resolution mass spectrometry: communicating confidence. *Environmental Science & Technology*, **48**(4): 2097-2098 (2014).

(5)	Qiu, J., F. Lü, T. Li, et al. A novel 4-set venn diagram model based on high-resolution mass spectrometry to monitor wastewater treatment. *Environmental Science & Technology*, **56**(20): 14753-14762 (2022).

(6)	Koch, B.P. and T. Dittmar. From mass to structure: an aromaticity index for high-resolution mass data of natural organic matter (vol 20, pg 926, 2006). *Rapid Communications in Mass Spectrometry*, **30**(1): 250-250 (2016).

(7)	Zhang, B.L., C. Shan, Z.N. Hao, et al. Transformation of dissolved organic matter during full-scale treatment of integrated chemical wastewater: Molecular composition correlated with spectral indexes and acute toxicity. *Water Research*, **157**: 472-482 (2019).

(8)	Wang, L., Y. Lin, J. Li, et al. Deciphering microbe-Mediated dissolved organic matter reactome in wastewater treatment plants using directed paired mass distance. *Environmental Science & Technology*, **58**(1): 739-750 (2024).

(9)	Frisch, M.J., G.W. Trucks, H.B. Schlegel, et al. Gaussian 16 Rev. A.03, (Wallingford, CT 2016).

(10)	Garza, A.J. and G.E. Scuseria. Predicting band gaps with hybrid density functionals. *Journal of Physical Chemistry Letters*, **7**(20): 4165-4170 (2016).

(11)	Witte, J., J.B. Neaton and M. Head-Gordon. Effective empirical corrections for basis set superposition error in the def2-SVPD basis: gCP and DFT-C. *Journal of Chemical Physics*, **146**(23). 234105 (2017).

(12)	Mörschel, P., J. Janikowski, G. Hilt, et al. Ligand-tuned regioselectivity of a cobalt-catalyzed diels-alder reaction.: A theoretical study. *Journal of the American Chemical Society*, **130**(28): 8952-8966 (2008).

(13)	Faza, O.N., R.A. Rodríguez and C.S. López. Performance of density functional theory on homogeneous gold catalysis. *Theoretical Chemistry Accounts*, **128**(4-6): 647-661 (2011).

(14)	Lu, T. and F.W. Chen. Multiwfn: A multifunctional wavefunction analyzer. *Journal of Computational Chemistry*, **33**(5): 580-592 (2012).

(15)	Zhang, J. and T. Lu. Efficient evaluation of electrostatic potential with computerized optimized code. *Physical Chemistry Chemical Physics*, **23**(36): 20323-20328 (2021).

(16)	Momma, K. and F. Izumi. VESTA 3 for three-dimensional visualization of crystal, volumetric and morphology data. *Journal of Applied Crystallography*, **44**: 1272-1276 (2011).
